# *Saccharomyces boulardii* CNCM I-745 and smectite treatment for pediatric acute gastroenteritis in China: a systematic review and meta-analysis

**DOI:** 10.3389/fped.2026.1747695

**Published:** 2026-02-19

**Authors:** Tong Li, Lynne Vernice McFarland

**Affiliations:** 1 Department of Gastroenterology, First Affiliated Hospital of Dalian Medical University, Dalian, China; 2 McFarland Consulting and Public Health Reserve Corp, Seattle, WA, United States

**Keywords:** acute pediatric diarrhea, clinical trials, meta-analysis, probiotic, smectite, *S. boulardii*

## Abstract

**Introduction:**

Pediatric acute gastroenteritis (PAGE) is a common cause of morbidity and mortality, especially among children under 5 years of age. Standard treatments typically include rehydration therapy, dietary modifications, antimicrobials, and adjunctive treatments with smectite or specific probiotics. The efficacy of adding *Saccharomyces boulardii* to standard treatments, including regimens that already incorporate smectites, remains not well known. Most trials evaluating this combination have been published in Chinese, which has limited global awareness of this type of treatment.

**Aim:**

This study aimed to meta-analytically examine whether the addition of *S. boulardii* CNCM I-745 to smectite is more effective in treating PAGE than smectite alone.

**Methods:**

Systematic searches were conducted in PubMed, Google Scholar, China National Knowledge Infrastructure, and the China Biology Medicine database up to 20 February 2025 receiving smectites. Eligible studies were randomized controlled trials conducted in China that compared *S. boulardii* CNCM I-745 with controls in children with PAGE receiving smectites, with no language restrictions. Data were independently extracted using standardized forms, including outcomes related to PAGE (cured, duration of PAGE, length of hospitalization, and immune markers) and potential confounding variables (dose, disease etiology).

**Results:**

Of 57 included trials (5,767 participants), *S. boulardii* CNCM I-745 significantly improved the cure rate (RR = 1.45, 95% CI 1.38, 1.53), reduced the duration of PAGE (SMD = −1.54 days, 95% CI −1.79, −1.29), improved the total effectiveness rating (RR = 1.21, 95% CI 1.18, 1.24), and reduced adverse events (RR = 0.64, 95% CI 0.43, 0.97).

**Conclusion:**

*S. boulardii* CNCM I-745 significantly improved cure rates, reduced the duration of PAGE, decreased stool frequency and vomiting, and shortened hospitalization duration, while being well-tolerated.

**Systematic Review Registration:**

http://www.crd.york.ac.uk/PROSPERO, PROSPERO #CRD42024567537.

## 1 Introduction

Pediatric acute gastroenteritis (PAGE) affects more than 1.7 billion children worldwide, with over 500,000 million deaths annually among children under 5 years of age (1–3). PAGE is associated with an increased risk of hospitalization and emergency department visits, increased risk of dehydration, and higher mortality (3, 4). The most common etiologies are viral (rotavirus and norovirus) and bacterial (*Escherichia coli* and non-typhoidal *Salmonella*) pathogens (5, 6).

Clinical guidelines for PAGE treatment, including the 2025 Chinese guideline and the European ESPGHAN guideline, recommend rehydration therapy, specific anti-infective medications (depending upon the type of etiology and severity of diarrhea), dietary adjustments, and the adjunctive use of either specific probiotics or smectite; however, these guidelines do not address the combined use of a probiotic in addition to smectite as a treatment strategy (7, 8).

Smectites (including diosmectite and montmorillonite) are a group of aluminum and magnesium silicate minerals commonly used to treat pediatric diarrhea. Their mechanisms of action include coating the intestinal mucosal surfaces to prevent pathogen invasion, absorbing toxins, enhancing IgA responses, binding mucin glycoproteins, and repairing intestinal mucosal damage. A study conducted in China found that adding a probiotic (“Bifid Triple Live”) to montmorillonite was more effective than montmorillonite alone (9).

As the intestinal microbiome is disrupted during PAGE, strategies aimed at restoring the microbiome have been widely investigated. Several guidelines evaluating probiotic use in PAGE have found some probiotic strains are effective, including *Saccharomyces boulardii* CNCM I-745, *Lacticaseibacillus* (*Lactobacillus*) *rhamnosus* GG, and *Limosilactobacillus* (*Lactobacillus*) *reuteri* DSM 17938, while other strains have not demonstrated effectiveness (10, 11). Reaching consensus on the most effective probiotic for PAGE has been challenging due to heterogeneity in study designs and conclusions based on pooled data from different types of probiotics, which do not account for the strain- and disease-specific effects of probiotics (12). *S. boulardii* CNCM I-745 is a widely available probiotic that has demonstrated effectiveness across a wide variety of intestinal disorders, including PAGE (13, 14). Its exact mechanisms of action are multifactorial and include interference with pathogen attachment, restoration of disrupted intestinal microbiota, inactivation of bacterial toxins [including those produced by *Vibrio cholera*, enterotoxigenic *E. coli* (ETEC), *Clostridioides difficile*, etc.], antisecretory effects via normalization of the transcellular transport of chloride, reduced loss of sodium and water, and immunomodulatory effects (15–17). In the last few years, several clinical trials conducted in China have been published but have not been included in non-Chinese systematic reviews and meta-analyses. Although a recent meta-analysis found significant efficacy of *S. boulardii* in the treatment of PAGE, it did not include trials evaluating the addition of *S. boulardii* to smectite therapy (18).

The aim of this meta-analysis was to systematically review the evidence from randomized controlled trials (RCTs) conducted in China on the adjunctive efficacy of adding *S. boulardii* to smectite for treating acute gastroenteritis in children.

## 2 Methods

### 2.1 Protocol

We conducted our meta-analysis following the Preferred Reporting Items for Systematic Reviews and Meta-Analyses (PRISMA 2020) guidelines (Supplementary Table S1) (19). The protocol was prospectively registered in the International Prospective Register of Systematic Reviews (PROSPERO # CRD42024567537; July 10, 2024; revised October 2024; available at http://www.crd.york.ac.uk/PROSPERO/).

### 2.2 Data sources

Publicly accessible databases, including Google Scholar, PubMed, the China National Knowledge Infrastructure, and the China Biology Medicine database (CMBdisc), were searched from database inception to 20 February 2025 to identify RCTs conducted in China for the treatment of PAGE comparing *S. boulardii* CNCM I-745 with controls in children who also received smectite. The search strategies are provided in Supplementary Table S2. No language restrictions were imposed, and non-English publications were translated. Recursive searches of the gray literature were performed by screening reference lists, authors, and reviews.

### 2.3 Study selection

The inclusion criteria were as follows: RCTs with prospective, parallel-group designs, participants randomized to either *S. boulardii* CNCM I-745 in combination with smectite (diosmectite or montmorillonite) or smectite alone (controls), children aged ≤18 years with acute diarrhea (defined as ≥3 loose or watery stools per day lasting <14 days), and living in China. All participants may receive standard therapies (oral or IV rehydration therapy, antiviral medications or antibiotics as needed, or dietary changes). Randomization was required to be clearly stated (not just “divided into two groups” or not specified). The strain had to be clearly identified as *S. boulardii* CNCM I-745, either by brand name (“Yihuo” or “Bioflor”), Biocodex import license number, or by listing Biocodex as the manufacturer. The probiotic had to be given orally at a designated daily dose for at least 3 days.

The exclusion criteria were as follows: adult patients (>18 years old); studies not conducted in China; non-human studies; case reports or case series; early phase 1 (safety) or phase 2 (mechanism of action, dose ranging, formulation, or pharmacokinetic) studies; retrospective case–control studies; studies without a control group; interventions that were not well-described; and reviews, meta-analyses, duplicate reports, or studies lacking original quantitative data. Trials that administered additional treatments, including specific antibiotics not directed at bacterial diarrhea, zinc, racedotril, fructose, aluminum diphosphate, other probiotics, or Chinese medicines, were also excluded. Studies involving other types of diarrhea (antibiotic-associated diarrhea, *C. difficile* infection, irritable bowel syndrome, diarrhea secondary to pneumonia, or allergic diarrhea) were also excluded.

### 2.4 Data extraction

Two reviewers (LM and TL) independently screened titles and abstracts and extracted data for 24 recommended items using a pre-designed data extraction form (18) following the standard methods for systematic reviews and meta-analyses (19–21). Any disagreements were resolved through discussion until a consensus was reached.

The data extracted included population, intervention, control, and outcome data (PICO): (1) population characteristics (pediatric, age range, and country), (2) intervention details (type of *S. boulardii*, daily dose, formulation, treatment duration, and follow-up period), (3) comparison groups (type of control group, including placebo-controlled or open-label, unblinded designs), and (4) outcomes, including improvement in PAGE symptoms (cure rate, duration of diarrhea, effectiveness rating, stool frequency/day by end of study), time to resolution of vomiting or fever, length of hospitalization, safety measures, and changes in immune markers. For data not reported in the published article, we attempted to contact the author or co-authors to obtain the missing information.

### 2.5 Risk of bias and strength of evidence

Each trial was reviewed for quality and risk of bias (RoB) and scored independently by both co-authors using standard methods (22). Study quality was assessed for the 24 recommended items for clinical trials and graded as high quality [≥18 items (75%) present], moderate quality (12–17 items present, at least 50%), and low quality (<12/24 items present). Risk of bias was assessed using the RoB 2.0 tool and was graded as low, some concerns, or high risk across five domains: randomization process, deviations from intended interventions, missing outcome data, measurement of outcomes, and selection of the reported result (22). The randomization process was rated as “low” if the randomization method was reported or if baseline characteristics were not significantly different. Deviations from intended interventions were rated as “low” when double- or single-blinding was used, “some concerns” when non-blinded controls were used but no deviations from group assignments were found, and “high” when open-label trials showed significant deviations from group assignments. Missing outcome data were scored based on attrition rates and rated as “low” for 0%–10% attrition, “some concerns” for 11%–50% attrition, and “high” for >50% attrition. Measurement of outcomes was rated as “low” when outcome assessments were single- or double-blinded or when outcomes in open-label trials were documented in inpatient medical records and “high” when unblinded trials were in outpatient settings or when patient status was not reported. Selection of the reported results was rated as “low” if outcomes were defined *a priori* and no *post hoc* outcomes were reported; otherwise, it was rated as “high” bias. A summary risk-of-bias figure was generated, and the impact of study quality was assessed (23). The strength and certainty of the evidence were assessed using the Grading of Recommendations, Assessment, Development and Evaluations tool (24).

### 2.6 Primary outcomes

The cure rate was defined as cessation of diarrhea (less than three loose or watery stools per day) by the end of the study intervention. The duration of diarrhea was defined as the number of days from enrollment to the first day of resolution of diarrhea symptoms. The total effective rate (TER), which measures the improvement of diarrheal symptoms, was categorized into three categories: “markedly effective or cured” (resolution of diarrhea symptoms by the end of the intervention), “effective or improved” (moderate improvement in symptoms), or “not effective” (persistent diarrhea symptoms at the end of the intervention). The total effectiveness rate was defined as the proportion of patients classified as either markedly effective or effective. Safety outcomes were defined as the occurrence of any adverse events reported during the study period.

### 2.7 Secondary outcomes

Other outcomes were also assessed and included time to resolution of vomiting or fever (defined as the number of days from study enrollment to the last day of symptoms), stool frequency (number of bowel movements per day at the end of treatment), length of hospitalization (mean duration of hospital stay among hospitalized patients), changes in cytokine levels from enrollment to the end of the study (CD4/CD8 ratio, TNF, CD3, CRP, interferon levels, IL-3, and IL-10), and changes in the intestinal microbiome from enrollment to the end of the study.

### 2.8 Subgroup analyses

Factors that might impact the efficacy of *S. boulardii* were also assessed, including the etiology of diarrhea (rotavirus, bacterial, or other causes), the daily dose of *S. boulardii*, the timing of intervention initiation (within 48 h of diarrhea onset or longer), risk of bias, and patient age group.

### 2.9 Data synthesis

Inclusion of studies in the meta-analysis required at least two RCTs using a common outcome measure that compared *S. boulardii* with a non-probiotic control. Statistical analyses and forest plot generation for pooled summary estimates were performed using Stata software version 16 (StataCorp, College Station, Texas) with the meta-analysis modules (25). Dichotomous outcomes were assessed using relative risks (RRs) with 95% confidence intervals (CIs), while continuous outcomes were evaluated using standardized mean differences (SMDs) with 95% CIs (26). The significance level was set at *p*-value <0.05. Heterogeneity across studies was evaluated using the I^2^ statistic (with values >50% indicating a high degree of heterogeneity). Bayesian random-effects models were used when heterogeneity was high (I^2^ > 50%); otherwise, fixed-effects models were used (26). Publication bias was assessed using funnel plots and Egger's test (25). Subgroup analyses were performed to explore sources of heterogeneity, which were assessed using the Cochrane *Q*-test (25). Sequential sensitivity analyses were performed to explore the extent to which outcomes depended on a particular trial. Trials with missing data for specific outcomes were excluded from the analyses of those respective outcomes.

## 3 Results

### 3.1 Literature search

**Figure 1:**
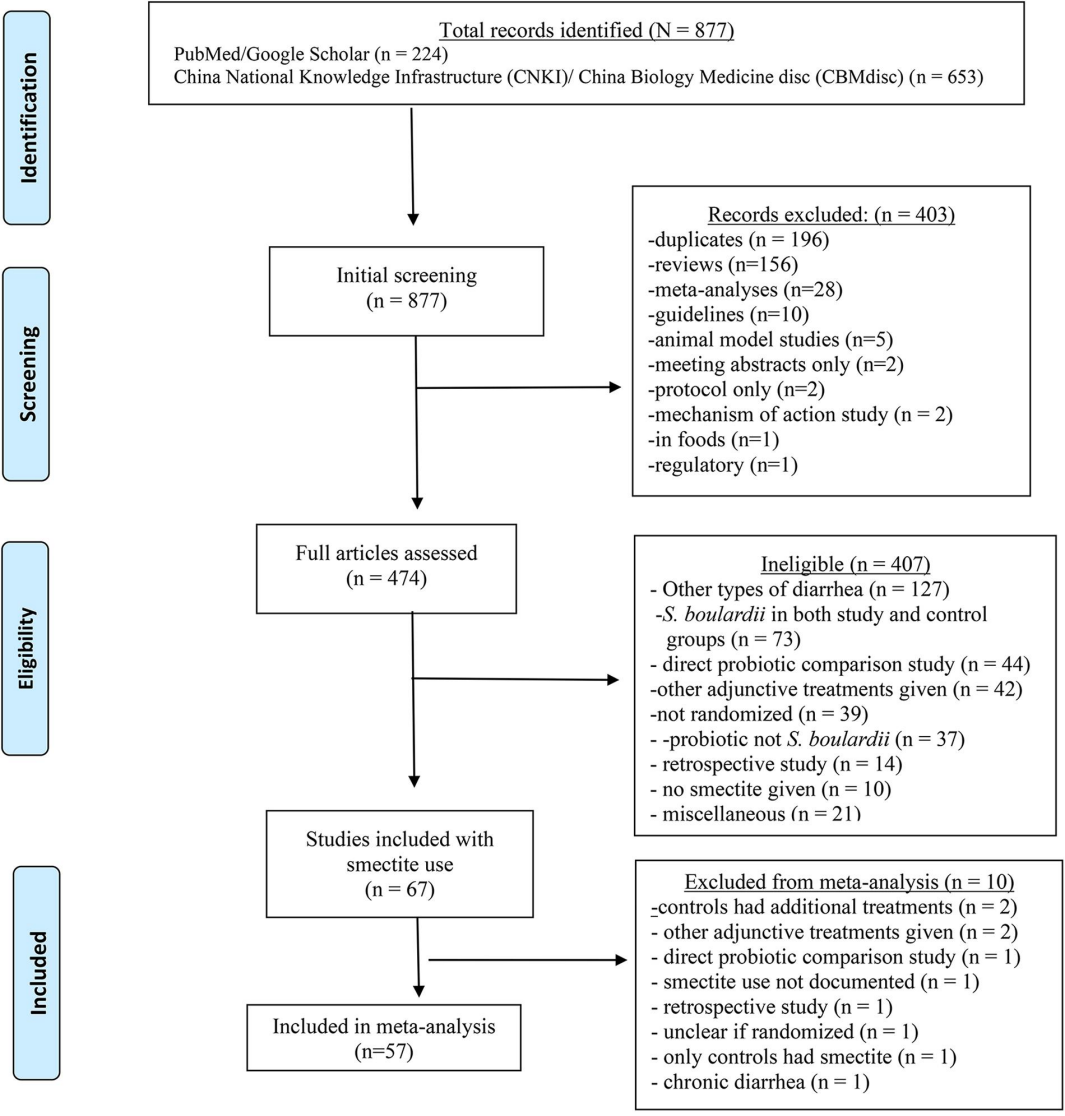
PRISMA study flowchart showing results of literature searches.

Our literature search screened 877 abstracts from database inception to 20 February 2025, of which 810 were excluded (Figure 1). Initial screening excluded non-RCT study designs or reviews/meta-analyses (*n* = 403). Full-text articles were then screened for eligibility, and 407 were excluded based on predefined exclusion criteria (most commonly due to inclusion of other types of diarrhea) (*n* = 127) or the use of *S. boulardii* in both intervention and control groups (*n* = 73). A total of 67 trials reported smectite use; however, after careful translation from Chinese into English and review by two researchers, 10 trials were excluded for failure to fulfil the inclusion criteria (Supplementary Table S3) (27–36). Ultimately, 57 randomized controlled trials (*N* = 5,767 participants) were included in the meta-analysis (37–93). Evidence of publication bias was found (Supplementary Figure S1), *P* < 0.001, suggesting the potential omission of small-scale trials with negative or non-significant results.

### 3.2 Study participant characteristics

The characteristics of the included trials and study participants are summarized in Supplementary Table S4. The RCTs enrolled children, either neonates (<1 month old) (43) or children aged 2 months to 10 years old; most trials enrolled patients shortly after the onset of diarrhea: 1–3 days (*n* = 34, 59.6%), 4–7 days (*n* = 11, 19.3%), and 8–14 days (*n* = 4,7%); however, 8 (14.0%) did not report when the timing of diarrhea onset. The most commonly identified etiology of PAGE was rotavirus (*n* = 25, 43.8%). One trial reported mixed viral etiologies (63), one reported bacterial etiologies (*Enterococcus*, *Salmonella*, or enteropathogenic *E*. *coli*) (74), and one reported a mix of bacterial, viral, and parasitic etiologies without specifying the types (86). Notably, 29 (50.9%) trials did not report etiological data. Most of the children were inpatients (*n* = 42, 73.7%). Only three trials enrolled outpatients (5.3%) (46, 58, 63), four enrolled a mix of inpatients and outpatients (7%) (37, 40, 57, 60), and eight trials (14%) did not report the status of enrolled children.

### 3.3 Study design

The study size ranged from 50 to 246 participants per trial (mean 101 ± 34 participants per trial). Most children (93%) received rehydration therapy. Some trials (*n* = 29, 50.9%) also allowed other treatments to be used in both groups as needed (antivirals, antibiotics, anti-inflammatory agents, or dietary changes). Two trials were single-blinded (patients) (62, 72), and none used a placebo control. Of the 57 trials, 12 (21%) had an overall “high” risk of bias, 43 (75.5%) were rated as having “some concerns” mainly due to the lack of blinding, and only two trials (3.5%) had a “low” risk of bias (Supplementary Figure S2). A review of 24 recommended factors for clinical trials (94) found that 55 studies (96.5%) reported 50%–70% of the factors, while only two studies (3.5%) reported >70% of the factors. The most common unreported factors included the method of randomization (36.8%) and adverse event or safety data (50.9%). The most common randomization method was the use of a random number table (*n* = 26); other methods (*n* = 10) included computer programs, lottery methods, card drawing, odd/even medical record numbers, or colored balls. Most trials reported no attrition or loss to follow-up (*n* = 54, 94.7%), while three trials reported low attrition rates (3%–8%) (38, 47, 48). Most trials did not follow children after discontinuation of the intervention (*n* = 53, 93%); however, four trials reported follow-up periods ranging from 2 days to 3 months post-intervention (45, 46, 68, 72). None of the trials reported sample size calculations.

### 3.4 Intervention characteristics

The *S. boulardii* strain was confirmed as CNCM I-745 when identified by brand name (Yihuo or Bioflor) in nine trials (15.8%), by import/registration number in 39 trials (68.4%), or by listing Biocodex as the manufacturer in nine trials (15.8%). Trials in which the strain could not be verified were excluded from our review. The duration of treatment ranged from 3 to 15 days in 45 RCTs (78.9%), extended to 12 weeks in one trial (64), and was not reported in 11 trials (19.3%) (Supplementary Table S4). The formulation of *S. boulardii* was as a powder in a sachet (*n* = 54, 94.7%), but it was not reported in three trials (5.3%) (60, 73, 76). The dose of *S. boulardii* varied across studies: 250 mg/day in seven trials (12.3%), 500 mg/day in one trial (1.8%), a range of 250–1,000 mg/day in one trial (84), and age-adjusted dosing in 48 trials (84.2%), typically ranging from 250 to 500 mg/day. Intervention initiation occurred within 48 h of diarrhea onset in 21 trials (36.8%), within 14 days in 28 trials (49.1%), and was not reported in eight trials (14%). The type of smectite was predominantly diosmectite (*n* = 53, 93%), while four trials used montmorillonite (7%) (40, 47, 68, 75).

### 3.5 Primary outcome: cure rates

**Figure 2:**
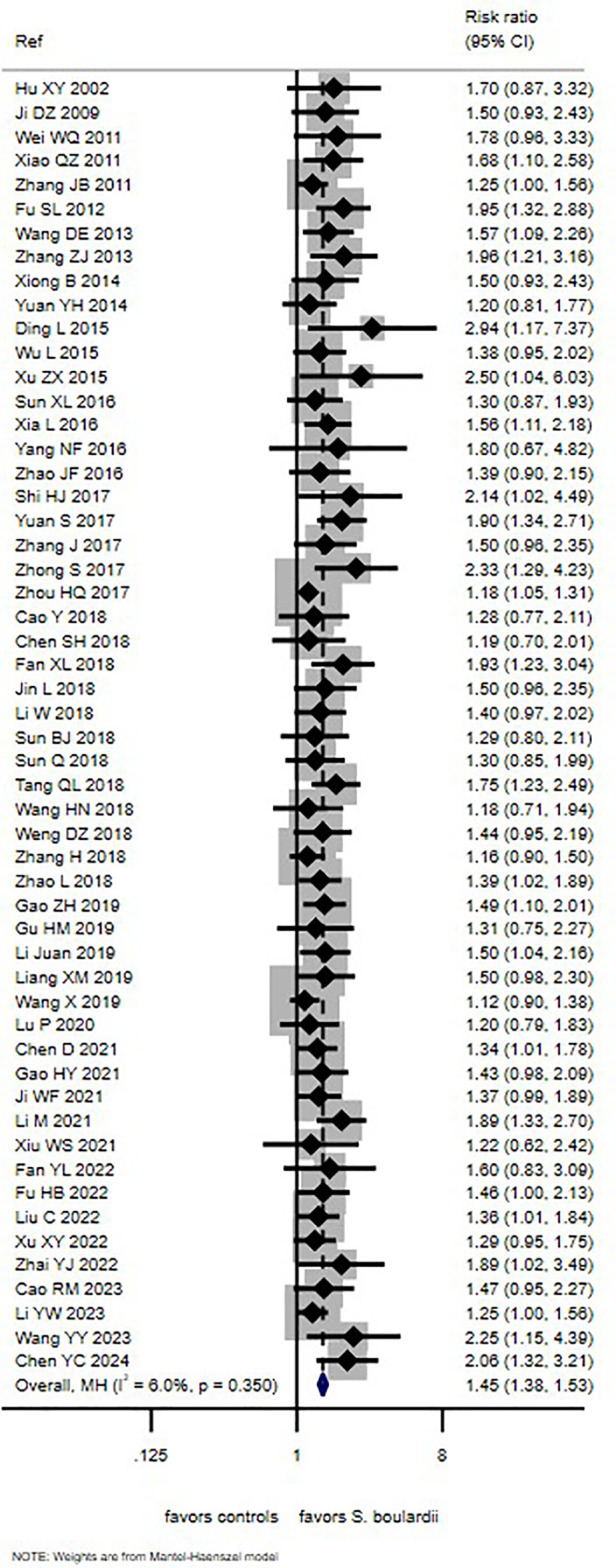
Forest plot of cure rates of PAGE comparing *S. boulardii* CNCM I-745 with controls.

Cure rates were reported in 54 trials (94.7%) but were not available in three trials (5.3%) (69, 70, 78). In the *S. boulardii* group, the cure rate ranged from 26.5% to 97.3%, while cure rates in controls ranged from 11.1% to 82.7% (Supplementary Table S5). The meta-analysis of pooled data from 54 trials showed a significantly higher cure rate with *S. boulardii* compared with control treatment (RR = 1.45, 95% CI 1.38, 1.53, I^2^ = 6%, *P* < 0.0001; Figure 2). Four factors had sufficient data to permit subgroup analyses (Supplementary Table S6), but none had a significant impact on efficacy. These factors included daily dose of *S. boulardii* (250 mg/day vs. age-adjusted dosing), etiology (rotaviral vs. other causes), or timing of treatment initiation (within 48 h vs. longer), and type of patient (inpatient, outpatient, or mixed).

### 3.6 Primary outcome: duration of PAGE

**Figure 3:**
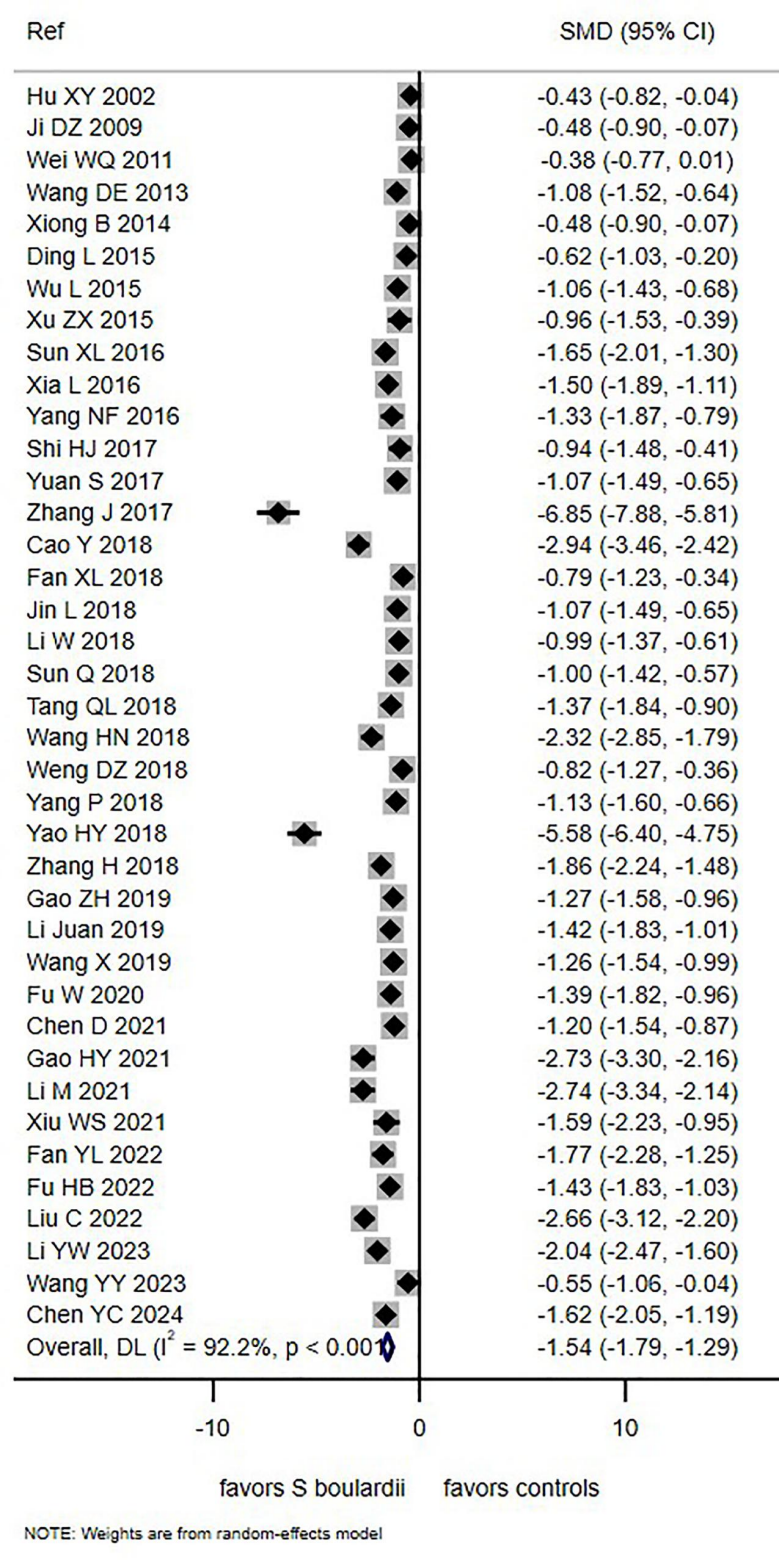
Forest plot of duration of PAGE diarrhea days comparing *S. boulardii* CNCM I-745 with controls.

The duration of diarrhea was reported in 39 trials (68.4%) but was not available in 18 trials (31.6%). The mean duration of diarrhea ranged from 1.2 to 5.7 days in children receiving *S. boulardii* and from 2.2 to 6.8 days in controls. Analysis of pooled data from 39 trials demonstrated that *S. boulardii* significantly reduced the duration of PAGE by 1.54 days (SMD: −1.54 days, 95% CI −1.79, −1.29, I^2^ = 92.2%, *P* < 0.0001; Figure 3). Sensitivity analysis excluding two trials with strong effects (56, 70) did not significantly change the outcome measurement (SMD = −1.34 days, 95% CI −1.53, −1.14, I^2^ = 77.3%, *P* < 0.0001). Factors that might reduce heterogeneity were explored using subgroup analyses, but they did not have a significant impact on this outcome (Supplementary Table S6). A daily dose of *S. boulardii* at 250 mg/day showed the greatest reduction in diarrhea duration (−2.20 days) compared to age-adjusted dosing (−1.47 days), but dose groups did not result in lower heterogeneity (97.1% and 91.3%, respectively). Among the 20 trials that reported diarrhea etiology, reduced heterogeneity was observed for rotaviral (86.7%) and other etiologies (57.5%), but the overall reduction in diarrhea duration was not significantly greater (SMD = −1.18 and −1.21 days, respectively), as shown in Supplementary Table S6. Timing of treatment initiation did not significantly reduce heterogeneity in the duration of diarrhea (started within 48 h, I^2^ = 92.9% or started after 48 h, I^2^ = 89%). The type of patient was assessed for inpatients; the duration was not significantly different in this group (SMD = −1.59 days, 95% CI −1.88, −1.29) nor was heterogeneity significantly reduced (I^2^ = 93.2%).

### 3.7 Primary outcome: total effectiveness rating

The TER was a common outcome in trials conducted in China and was reported in 53 trials (93.0%) (Supplementary Table S5). The TER ranged from 83.7% to 98.4% in the *S. boulardii* groups and was lower in the controls (63.3%–86.7%). Analysis of pooled data from the 53 trials demonstrated that *S. boulardii* had a significantly higher TER compared with controls (RR = 1.21, 95% CI 1.18, 1.24, I^2^ = 0%, *P* < 0.0001; Table 1). Subgroup analyses based on daily dose, diarrhea etiology, timing of treatment initiation, and type of patient did not have a significant impact on this outcome (Supplementary Table S6).

**Table 1:** Outcomes comparing *S. boulardii* CNCM I-745 with controls for the treatment of PAGE.

Outcome	Measure	Number of RCTs	Overall effect
Primary outcomes	Cured	54	RR = 1.45 (1.38, 1.53) * I^2^ = 6.0%
	Duration of PAGE (days)	39	SMD = −1.54 (−1.79, −1.29) * I^2^ = 92.2%
	Total effective rate	53	RR = 1.21 (1.18, 1.24) * I^2^ = 0%
	Adverse event frequency	11	RR = 0.64 (0.43, 0.97) * I^2^ = 0%
Secondary outcomes	Daily BM/day	26	SMD = −1.76 (−2.19, −1.34) * I^2^= 95.8%
	Vomiting cessation (days)	16	SMD = −1.75 (−2.27, −1.23) * I^2^= 95.3%
	Length of hospitalization (days)	11	SMD = −1.68 (−2.19, −1.16) * I^2^ = 92.9%
	TNF-α	10	SMD = −2.42 (−3.23, −1.60) * I^2^ = 96.5%
	CD4/CD8 ratio	16	SMD = 0.57 (−0.24, 1.36) I^2^ = 97.8%

* *P* < 0.001.

BM, bowel movements; PAGE, pediatric acute gastroenteritis; RCTs, randomized controlled trials; RR, relative risk; SMD, standardized mean difference; TNF-α, tumor necrosis factor (pg/mL).

### 3.8 Primary outcome: adverse events

**Figure 4:**
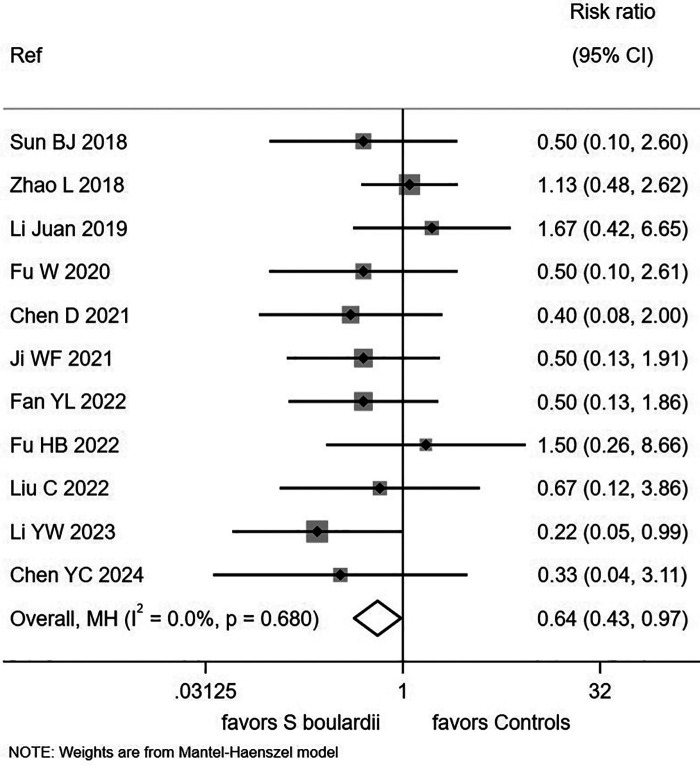
Forest plot of adverse event frequency comparing *S. boulardii* CNCM I-745 with controls.

Of the 57 included trials, 28 (49.1%) reported safety data. Among these, 15 trials (26.3%) explicitly stated that no adverse events occurred, while 13 trials (22.8%) reported at least one adverse event. The remaining 29 trials (50.9%) did not report any safety data (Supplementary Table S4). Of the 13 RCTs reporting adverse events, 11 provided data stratified by study group, while two trials did not (52, 77). Children receiving *S. boulardii* had a significantly reduced rate of adverse events (RR = 0.64, 95% CI 0.43, 0.97, I^2^ = 0%, *P* = 0.03; Figure 4). No significant differences were observed in the types of adverse events reported between the two groups.

### 3.9 Secondary outcomes

Daily stool frequency, duration of vomiting, and length of hospitalization were also analyzed. Stool frequency (bowel movements per day) was reported in 26 trials (45.6%) . In the *S. boulardii* groups, stool frequency ranged from 1.1 to 3.2 bowel movements per day, whereas controls reported higher frequencies (ranging from 1.5 to 5.5 bowel movements per day) (Supplementary Table S7). Pooled data analysis found a significant reduction in stool frequency when *S. boulardii* was given (SMD = −1.76/day, 95% CI −2.19, −1.34, I^2^ = 95.8%, *P* < 0.001; Table 1). Vomiting, a marker of severe dehydration, was reported in only 16 trials (28.1%). The duration of vomiting ranged from 1 to 2 days in the *S. boulardii* groups and from 1 to 4 days in controls. A meta-analysis found that *S. boulardii* significantly reduced the duration of vomiting (SMD = −1.75 days, 95% CI −2.27, −1.23, I^2^ = 95.3%, *P* < 0.0001; Table 1). Length of stay was reported in 11 trials (19.3%) (Supplementary Table S7). Children were hospitalized for 4–6 days in the *S. boulardii* group and 6–7 days in the controls. A meta-analysis found that *S. boulardii* significantly reduced the length of hospitalization by nearly 2 days (SMD = −1.68 days, 95% CI −2.19, −1.16, I^2^ = 92.9%, *P* < 0.0001; Table 1). Subgroup analyses did not find any factors that significantly reduced heterogeneity; heterogeneity persisted across all examined subgroups, including rotavirus etiology (I^2^ = 88.4%), age-adjusted dosing (I^2^ = 93.8%) or fixed dosing of 250 mg/day (I^2^ = 86.4%), initiation of treatment within 48 h (I^2^ = 96.2%) or after 48 h (I^2^ = 93.4%), and inpatients (I^2^ = 92.9%).

**Figure 5:**
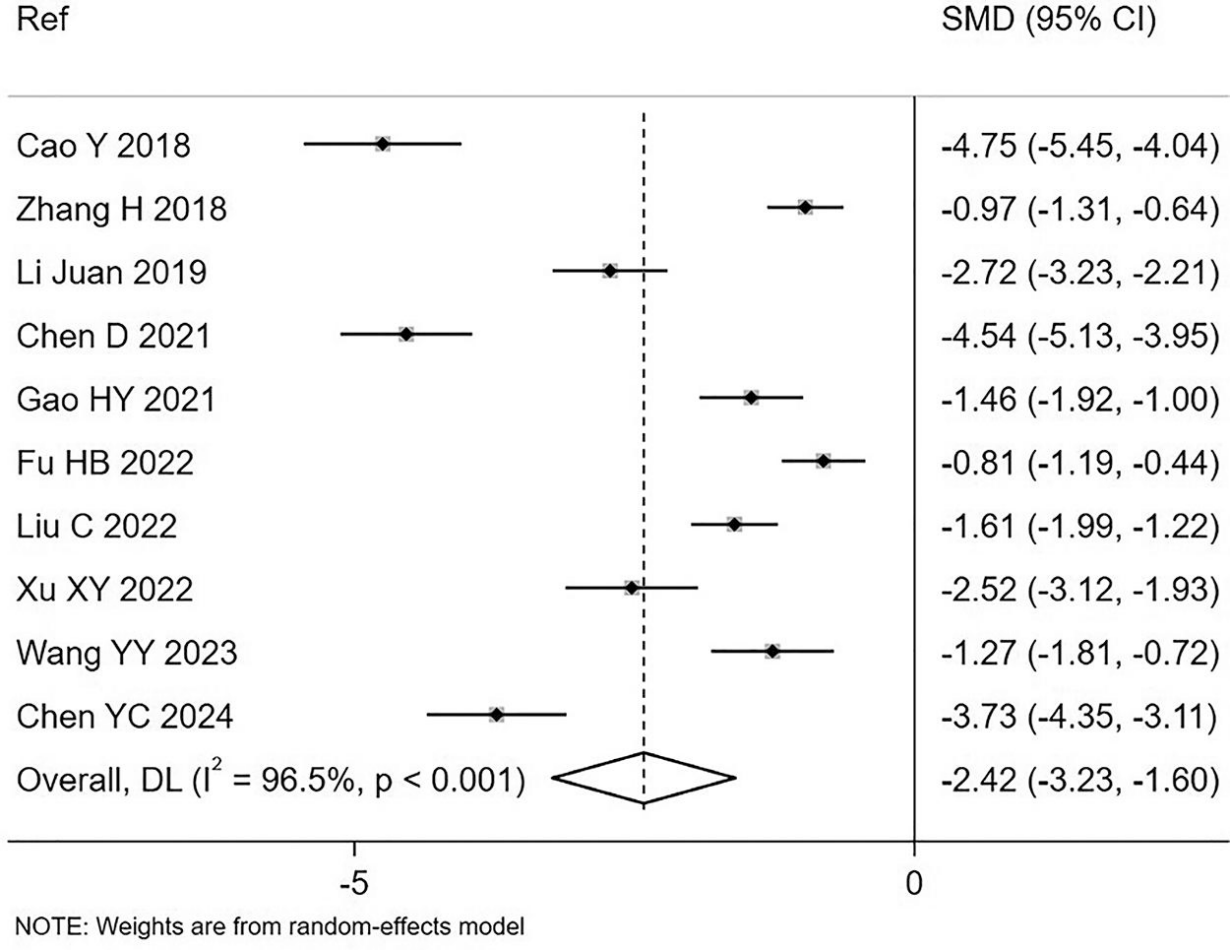
Forest plot of change in TNF levels from enrollment to the end of treatment by study groups comparing *S. boulardii* CNCM I-745 with controls.

Changes in immune markers were also analyzed (Supplementary Table S8). Changes in TNF-α were reported in 10 trials (17.5%). A meta-analysis revealed a significant reduction in TNF-α levels (SMD = −2.42, 95% CI −3.23, −1.60, I^2^ = 96.5%, *P* < 0.0001; Figure 5). Given the high heterogeneity, an analysis was conducted to identify factors that might reduce it, but none were identified. Change in TNF-α levels were similar for rotaviral diarrhea (SMD = −2.12, 95% CI −2.91, −1.33, I^2^ = 93.9%), age-adjusted dosing (SMD = −2.42, 95% CI −3.23, −1.6, I^2^ = 96.5%), and initiation of treatment (within 48 h: SMD = −2.32, 95% CI −3.85, −0.79, I^2^ = 97.4% or after 48 h: SMD = −2.48, 95% CI −3.54, −1.43, I^2^ = 96.5%). All these trials were in inpatients. Changes in the CD4/CD8 ratio during the study were reported in 17 trials (29.8%), while 40 trials (70.2%) did not report this outcome. No significant difference in CD4/CD8 ratios was observed between the study groups (Table 1). Thirteen trials reported CD3 data, and pooled analysis showed that *S. boulardii* significantly increased the levels of CD3 compared with controls (SMD = 1.90, 95% CI 1.4, 2.4, I^2^ = 92.9%, *P* < 0.0001). Changes in other immune markers (CRP, IL-10, IL-3) were not reported by sufficient numbers of trials to allow meaningful analysis.

Other outcomes were infrequently reported and thus could not be robustly analyzed, including reductions in abdominal pain (reported in eight trials), changes in the intestinal microbiome (reported in five trials), gastrointestinal (GI) hormone levels (reported in two trials) (82, 90), and intestinal barrier markers (reported in one trial) (93).

## 4 Discussion

Our study found that the addition of *S. boulardii* CNCM I-745 to smectite resulted in a significantly reduced duration of PAGE and shortened length of hospitalization in trials conducted in China. *S. boulardii* and smectite provided additional clinical benefits that extended beyond primary outcome goals (reducing the duration of diarrhea and length of hospital stay) mentioned in clinical guidelines (7, 8). The combination of *S. boulardii* with smectite significantly improved cure rates, improved the total effectiveness rate (which reflects improvements in diarrheal symptoms), decreased daily stool frequency by the end of treatment, and enhanced immune responses. To our knowledge, this is the first meta-analysis to report the efficacy of *S. boulardii* CNCM I-745 in combination with smectite compared with smectite controls for the treatment of PAGE.

Rehydration therapy is the first-line treatment for PAGE, but it does not reduce the duration of diarrhea nor improve stool frequency. Adjunctive therapies, such as smectite or certain probiotics, are commonly added to rehydration treatment to improve diarrheal symptoms (7, 8). Evidence from two meta-analyses revealed that when smectite is given with rehydration therapy, the duration of diarrhea was reduced by 22.7 h (based on six trials) (94) or 24.4 h (based on 18 trials) (95), but neither review assessed the addition of probiotics. Specific strains of probiotics (*S. boulardii*, *L. rhamnosus* GG, *L. reuteri*, *Bifidobacterium lactis*, or *Bacillus clausii*), when added to rehydration therapy, have also been found to effectively improve PAGE, but these trials did not include smectite use (14, 18, 96). Six meta-analyses found that when *S. boulardii* was added to rehydration therapy, a significant reduction in the duration of diarrhea was observed (ranging from 0.72 to 1.63 days), but these trials did not use smectite (14, 18, 97–100).

Evidence from individual trials may indicate potential probiotic candidates for the treatment of PAGE. In four randomized direct-comparison trials, *S. boulardii* CNCM I-745 was found to reduce the duration of PAGE by 0.5–1 day compared with multi-strain probiotic formulations, including *Lactobacillus bulgaricus* and *Streptococcus thermophilus* (101), “Bifido Quad Viable” (*Bifidobacterium infantis*, *Lactobacillus acidophilus*, *Bacillus cereus*, *Streptococcus faecalis*) (102), “Golden Bifid” (*Bifidobacterium longum*, *L. bulgaricus*, *S. faecalis*) (103), or “Enterogermina” (four *Bacillus clausii* strains O/C, SIN, N/R, and T) (104). Wei et al. (105) compared the combination of *L. acidophilus* and montmorillonite with montmorillonite controls and found a significant reduction in the duration of diarrhea by 1.19 days. In contrast, Pieścik-Lech et al. (106) did not find a significant decrease in the duration of PAGE when the combination of *L. rhamnosu*s GG and smectite was compared with *L. rhamnosus* alone. None of our included trials compared the combination of *S. boulardii* with smectite to *S. boulardii* alone.

Our analysis also investigated the interaction between *S. boulardii* and the immune system. Acute diarrhea is related to changes in cellular immune function; thus, components of T-cell subsets, such as CD3 and CD4/CD8 ratios, may be useful for monitoring the response to therapies. Although *S. boulardii* showed a significant reduction in TNF-α levels and an increase in CD3 levels, no significant differences were found for CD4/CD8 levels. Other immune markers (IL-6, IL-10, etc.) may also be influenced by *S. boulardii* but were not reported in most trials.

Although many trials did not report adverse event data, 54% of those that did reported no adverse events during their trial. This finding is consistent with previous reviews demonstrating that *S. boulardii* is well tolerated (13, 14).

Our meta-analysis has several strengths. One strength of this meta-analysis was the robust findings based on a substantial number of RCTs. We were able to control for probiotic strain-specificity by limiting inclusion to trials using only one strain (*S. boulardii* CNCM I-745). Trials that did not clearly describe the strain of *S. boulardii* used were excluded to prevent bias. Trials with other types of supplements added to probiotics for the treatment of PAGE (such as zinc, antibiotics, and racecadotril) were excluded. Use of rigorous inclusion and exclusion criteria resulted in trials with an overall low risk of bias or with some concerns of bias. All potential biases led to the use of open, unblinded controls. The strength of the recommendation for *S. boulardii* for the treatment of PAGE was rated as moderate to high (Supplementary Table S9).

Limitations of our review include the fact that most trials used open-label controls and only two trials were single-blinded. We could not include more than 20 trials because they did not identify the *S. boulardii* strain used. We were limited in our safety analysis, as 51% of the trials did not report any adverse events. Some outcomes involved in the pathogenesis of PAGE, such as gastrointestinal (GI) hormones and barrier markers, were infrequently reported and thus could not be assessed. We could not identify the sources of the high heterogeneity observed across the 57 trials for three outcomes (duration of diarrhea, length of hospital stay, and TNF-α levels). Subgroup analyses based on reported factors, such as daily dose, etiology, timing of treatment initiation, or type of patient, did not reduce the degree of heterogeneity. However, the cure rate and total effectiveness rating demonstrated low heterogeneity. Another limitation was the time required to translate the Chinese articles to enable data analysis (total time 7 months). As other trials may have been published since the last literature review in February 2025, we screened the literature through December 2025 but found only two recently published trials (107, 108). The inclusion of these two new trials did not significantly change the results of these analyses; therefore, they were not included.

The generalization of these results should be viewed with caution, as all included trials were conducted in China. However, trials conducted in other countries show comparable efficacy of *S. boulardii* (Supplementary Figure S3) (13, 14). Szajewska et al. (14) compared 13 RCTs of *S. boulardii* from different countries, including Argentina, Bolivia, India, Italy, Indonesia, Turkey, and Pakistan, and found that *S. boulardii* was equally effective in low- to moderate-index and high-index countries; however, none of these trials were conducted in China. McFarland and Li analyzed 10 trials conducted in China evaluating *S. boulardii* without smectite and also found a significant reduction in the duration of PAGE by 1 day (18).

Clinical implications of this review for clinical practice and policy indicate that *S. boulardii* CNCM I-745 can be safely and effectively combined with smectite to treat children with PAGE and may reduce healthcare costs by shortening the length of hospitalization for inpatients.

Future meta-analyses of combining probiotics with supplemental therapies need to account for probiotic strain-specificity to support any valid conclusions (21). The low-to-moderate quality of these trials was typically due to non-adherence to CONSORT guidelines for reporting clinical trials (20). Future trials need to follow these recommendations more closely by using blinded interventions (study staff and study participants), using placebo with standard treatments to reduce possible bias, completely describing the randomization method, stating how allocation assignments were kept blinded and using standardized primary outcome measures (as multiple outcomes were described), reporting sample size calculations, and completely identifying the *S. boulardii* strain used. The role of GI hormones and barrier factors should be investigated in future studies.

## 5 Conclusions

This is the first comprehensive analysis of clinical trials using *S. boulardii* CNCM I-745 as an adjunct to smectite for the treatment of children with acute diarrhea, a common clinical practice in China. *S. boulardii* CNCM I-745 significantly improved cure rates, reduced the duration of PAGE, decreased stool frequency and vomiting, and shortened hospitalization duration, while being well-tolerated.

## Data Availability

The original contributions presented in the study are included in the article/Supplementary Material, further inquiries can be directed to the corresponding author.

## References

[1] Troeger CE Khalil IA Blacker BF Biehl MH Albertson SB Zimsen SR Quantifying risks and interventions that have affected the burden of diarrhoea among children younger than 5 years: an analysis of the global burden of disease study 2017. Lancet Infect Dis. (2020) 20:37–59. 10.1016/S1473-3099(19)30401-3 31678029 PMC7340495

[2] Aghsaeifard Z Heidari G Alizadeh R. Understanding the use of oral rehydration therapy: a narrative review from clinical practice to main recommendations. Health Sci Rep. (2022) 5:e827. 10.1002/hsr2.827 36110343 PMC9464461

[3] World Health Organization. Diarrhoeal disease (2024). p. 1. Available online at: https://www.who.int/news-room/fact-sheets/detail/diarrhoeal-disease (Accessed June 3, 2025).

[4] Zhang SX Zhou YM Xu W Li-Guang T Chen JX Chen SH Impact of co-infections with enteric pathogens on children suffering from acute diarrhea in southwest China. Infect Dis Poverty. (2016) 5:1–13. 10.1186/s40249-016-0157-2 27349521 PMC4922062

[5] Wang LP Zhou SX Wang X Lu QB Shi LS Ren X Etiological, epidemiological, and clinical features of acute diarrhea in China. Nature Comm. (2021) 12:1–12. 10.1038/s41467-021-22551-z PMC8085116 33927201

[6] Zhou HL Bessey T Wang SM Mo ZJ Barclay L Wang JX Burden and etiology of moderate and severe diarrhea in children less than 5 years of age living in north and south of China: prospective, population-based surveillance. Gut Pathog. (2021) 13(1):33. 10.1186/s13099-021-00428-2 34030738 PMC8142869

[7] Fang YH Wan CM Gong ST Fang F Sun M Qian Y Clinical practice guidelines for acute infectious diarrhea in children in China (2024). World J Pediatr. (2025) 71:708–19. 10.1007/s12519-025-00894-7 40437180

[8] Guarino A Ashkenazi S Gendrel D Berkely JA Boey C Bruzzese D European Society for Pediatric Gastroenterology, Hepatology, and Nutrition/European Society for Pediatric Infectious diseases evidence-based guidelines for the management of acute gastroenteritis in children in Europe. J Ped Gastroentero Nutr. (2014) 59(1):132–52. 10.1097/MPG.0000000000000375 24739189

[9] Zhang L Dong HF Dong CL Huaizhen X Lan M. Clinical observations on the combination of probiotics and montmorillonite in the treatment of pediatric acute diarrhea. Chin J Gen Pract. (2008) 6(8):793–4.

[10] Szajewska H Canani RB Domellöf M Guarino A Hojsak H Indrio F Probiotics for the management of pediatric gastrointestinal disorders: position paper of the ESPGHAN special interest group on gut microbiota and modifications. J Ped Gastroenterol Nutri. (2023) 76:232–47. 10.1097/MPG.0000000000003633 36219218

[11] Chen J Cheng X Hua Z Huang Y Huang Z Li X Evidence-based guideline for clinical application of probiotics in pediatrics (2023). Chinese J Pract Ped. (2024) 39(01):1–15. 10.19538/j.ek2024010601

[12] McFarland LV Evans CT Goldstein EJ. Strain-specificity and disease-specificity of probiotic efficacy: a systematic review and meta-analysis. Front Med. (2018) 5:124. 10.3389/fmed.2018.00124 PMC5949321 29868585

[13] McFarland LV. Systematic review and meta-analysis of Saccharomyces boulardii in adult patients. World J Gastroenterol. (2010) 16:2202–22. 10.3748/wjg.v16.i18.2202 20458757 PMC2868213

[14] Szajewska H Kołodziej M Zalewski BM. Systematic review with meta-analysis: Saccharomyces boulardii for treating acute gastroenteritis in children-a 2020 update. Aliment Pharmacol Ther. (2020) 51:678–88. 10.1111/apt.15659 32056266

[15] Czerucka D Rampal P. Diversity of Saccharomyces boulardii CNCM I-745 mechanisms of action against intestinal infections. World J Gastroenterol. (2019) 25(18):2188–203. 10.3748/wjg.v25.i18.2188 31143070 PMC6526157

[16] Plaza-Diaz J Ruiz-Ojeda FJ Gil-Campos M Gil A. Mechanisms of action of probiotics. Adv Nutr. (2019) 10(Suppl 1):S49–66. 10.1093/advances/nmy063 30721959 PMC6363529

[17] Monjaraz EM Arellano KR Mayer AL Palacios-Gonzolez B Bustamante RC Mayans JA. Gut microbiota in Mexican children with acute diarrhea: an observational study. Ped Infect Dis J. (2021) 40(8):704–9. 10.1097/INF.0000000000003128 34250970

[18] McFarland LV Li T. Efficacy and safety of Saccharomyces boulardii CNCM I-745 for the treatment of pediatric acute diarrhea in China: a systematic review and meta-analysis. Front Cell Infect Microbiol. (2025) 15:1587792. 10.3389/fcimb.2025.1587792 40535538 PMC12174131

[19] Page MJ McKenzie JE Bossuyt PM Boutron I Hoffmann TC Mulrow CD The PRISMA 2020 statement: an updated guideline for reporting systematic reviews. Br Med J. (2021) 372:n160. 10.1136/bmj.n160 33782057 PMC8005924

[20] Moher D Hopewell S Schulz KF Montori V Gøtzsche PC Devereaux PJ CONSORT 2010 Explanation and elaboration: updated guidelines for reporting parallel group randomised trials. Br Med J. (2010) 340:c869. 10.1136/bmj.c869 20332511 PMC2844943

[21] McFarland LV Hecht G Sanders ME Goff DA Goldstein EJ Hill C Recommendations to improve quality of probiotic systematic reviews with meta-analyses. JAMA Network Open. (2023) 6:e2346872. 10.1001/jamanetworkopen.2023.46872 38064222

[22] Sterne JA Savović J Page MJ Elbers RG Blencowe NS Boutron I Rob 2: a revised tool for assessing risk of bias in randomised trials. Br Med J. (2019) 366:14898. 10.1136/bmj.14898 31462531

[23] McGuinness LA. Robvis: An R package and web application for visualising risk-of-bias assessments. Available online at: https://github.com/mcguinlu/robvis (Accessed May 20, 2024). 10.1002/jrsm.1411 32336025

[24] Guyatt GH Oxman AD Schünemann HJ Tugwell P Knottnerus A. GRADE guidelines: a new series of articles in the Journal of Clinical Epidemiology. J Clin Epidemiol. (2011) 64(4):380–2. 10.1016/j.jclinepi.2010.09.011 21185693

[25] Palmer TM Sterne JAC. Meta-analysis in Stata: An Updated Collection from the Stata Journal. 2nd edn College Station, Texas: Published by Stata Press (2016).

[26] Borenstein M Hedges LV Higgins JP Rothstein HR. A basic introduction to fixed-effect and random-effects models for meta-analysis. Res Syn Methods. (2010) 1:97–111. 10.1002/jrsm.12 26061376

[27] Feng NC Lei ZX Yang H Hu L. Clinical efficacy of Saccharomyces boulardii combined with mezlocillin in the treatment of children with infectious diarrhea and effect on serum CRP, PCT and IL-8. Chinese J Integr Tradit West Med Dig. (2018) 26(2):194–7.

[28] Huang K. Efficacy of Saccharomyces boulardii in the treatment of pediatric acute diarrhea and its impact on cellular immune function. North Pharmaceut J. (2018) 15(4):147–61.

[29] Liu T. Analysis of the clinical effect of treating children with rotavirus gastroenteritis with Saccharomyces boulardii and Bifidobacterium quadruple live bacteria. Cardiovasc Dis Integr Tradit Chinese West Med. (2020). 8(34):57–63. 10.16282/j.cnki.cn11-9336/r.2020.34.040

[30] Liu Z. Analysis of the efficacy of Saccharomyces boulardii powder in the treatment of pediatric acute diarrhea. J Med Theor Prac. (2019) 32(21):3512–3. 10.19381/j.issn.1001-7585.2019.21.059

[31] Shi F. Analysis of the efficacy of S. boulardii in treating acute diarrhea in children. Jilin Med J. (2015) 36(11):9522.

[32] Wang D Cheng Y Zhou H. Effects of Saccharomyces boulardii combined with montmorillonite powder on acute diarrhea in children and T-cell subsets. Drug Eval Res. (2020) 43(6):1095–8. 10.7501/j.issn.1674-6376.2020.06.020

[33] Wang WH. Cinical research on the prevention and treatment of acute diarrhea in children with Saccharomyces boulardii. J Prev Med Chinese People’s Lib Army. (2016) 34(4):302. 10.13704/j.cnki.jyyx.2016.s2.273

[34] Zhang M Chen H. Clinical efficacy of Saccharomyces boulardii in the treatment of infantile rotavirus enteritis. Pract Med J. (2014) 30(22):3698–9. 10.3969/j.issn.1006-5725.2014.22.059

[35] Zhang XQ Hu WH. Effect of Saccharomyces boulardii on serum cytokines and clinical efficacy in infantile rotavirus enteritis. Chin Remedies & Clinics. (2014) 14(1):72–3. 10.11655/zgywylc.2014.01.031

[36] Zou J. Efficacy of montmorillonite powder combined with Saccharomyces boulardii in the treatment of pediatric acute diarrhea. Pract Clin Med. (2018) 19:63–4. 10.13764/j.cnki.lcsy.2018.03.023

[37] Hu XY Shi L Ruan GT. Clinical study of treatment for infant rotavirus enteritis by Saccharomyces boulardii. Chin J Microecol. (2002) 14(6):42–3. 10.13381/j.cnki.cjm.2002.06.019

[38] Ji DZ Zou SQ Yuan LY. Saccharomyces boulardii in the treatment of acute diarrhea in children: a randomized placebo-controlled trial. J Ped Pharm. (2009) 15(1):13–5. 10.13407/j.cnki.jpp.1672-108X.2009.01.016

[39] Wei WQ Lu HJ. Clinical exploration of Saccharomyces boulardii in the treatment of infantile rotavirus enteritis. Chin J Primary Med Pharm. (2011) 18(10):1376–7. 10.3760/cma.j.issn.1008-6706.2011.10.045

[40] Xiao QZ. Observation on the effect of Saccharomyces boulardii in treating infantile rotavirus enteritis. J Nantong Univ (Medical Sciences). (2011) 31(5):372–3.

[41] Zhang JB Lu JH Zhao GF. Effect observation of the Saccharomyces boulardii (S. boulardii) sachets in the treatment of children with acute diarrhea. Chin J Clinical Rational Drug Use. (2011) 4(5B):23–4.

[42] Fu S. Effects of Saccharomyces boulardii combined with smectite in treating children with acute diarrhea. China Mod Doctor. (2012) 50(21):68–70.

[43] Wang D. Observation on the efficacy of Saccharomyces boulardii in the treatment of neonatal rotavirus enteritis. Mod Prac Med. (2013) 25(11):1218–9. 10.3969/j.issn.1671-0800-2013.11.008

[44] Zhang ZJ Wang P. Observation on the efficacy of Saccharomyces boulardii in the treatment of infantile diarrhea. J Pract Med Techniques. (2013) 20(5):545–6.

[45] Xiong B. Observation on the efficacy of Saccharomyces boulardii in the treatment of rotavirus enteritis. Clin Educ Gen Pract. (2014) 12(2):206–8. 10.13558/j.cnki.issn1672-3686.2014.02.035

[46] Yuan YH. Effect of Saccharomyces boulardii combined with smectite on cellular immunity and efficacy in children with rotavirus enteritis. Chin J Microecol. (2014) 26(3):310–2. 10.13381/j.cnki.cjm.201403015

[47] Ding L Zhu J Gu XY Zhou H Lin X Wang Z Clinical study of Saccharomyces boulardii in the treatment of pediatric rotavirus enteritis. J Nantong Univ (Medical Sciences). (2015) 35(1):70–2.

[48] Wu LQ Chen JP He NH. Efficacy comparison between Saccharomyces boulardii sachets and tetragenous viable bifidobacterium tablets in the treatment of rotaviral gastroenteritis. Chongqing Med J. (2015) 44(31):4349–51. 10.3969/j.issn.1671-8348.2015.31.010

[49] Xu ZX Qian BM. The clinical effect observation of S. boulardii scattered combined with diosmectite in diarrhea in children. Chin J Clinic Rational Drug Use. (2015) 8(4):27–8. 10.15887/j.cnki.13-1389/2015.10.016

[50] Sun XL. Efficacy of S. boulardii in the treatment of acute pediatric diarrhea and its effect on cellular immune function. Chin Remedies Clinics. (2016) 16(7):1029–31. 10.11655/zgywylc2016.07.04

[51] Xia L. The clinical efficacy of Saccharomyces boulardii powder in treatment of infantile rotavirus enteritis. J Clin Pathol Res. (2016) 36(10):1487–91. 10.3978/j.issn.2095-6959.2016.10.004

[52] Yang NF Fan HS Wu J. Clinical efficacy and safety of S. boulardii powder combined with diosmectite powder in treatment of acute diarrhea in children. Chin J Biochemical Drugs. (2016) 36(6):179–81.

[53] Zhao JF Zhao LM. Clinical effect of S. boulardii sachets in the treatment of children with acute diarrhea and its impact on the cellular immune function. Chin Ped Integrat Trad Western Med. (2016) 8(6):589–91. 10.3969/j.issn.1674-3865.2016.06.012

[54] Shi HJ. Clinical curative effect and safety of S. boulardii sachets combined with diosmectite powder in the treatment of children with acute diarrhea. Chin J Modern Drug Appl. (2017) 11(7):91–3. 10.14164/j.cnki.cn11-5581/r.2017.07.044

[55] Yuan SQ Liu CH Wang X. Clinical efficacy of Saccharomyces boulardii in treating children with acute diarrhea. China Pharm. (2017) 26(11):55–7. 10.3969/j.issn.1006-4931.2012.11.018

[56] Zhang J Chen HY. Clinical observation on the therapeutic effect of S. boulardii combined with diosmectite powder in children with acute diarrhea. Henan Med Res. (2017) 26(20):3771–2. 10.3969/j.issn.1004-437X.2017.20.078

[57] Zhong S. Analysis of the therapeutic effect of S. boulardii on acute pediatric diarrhea and its impact on cellular immune function. Chin J Modern Drug Appl. (2017) 11(20):122–3. 10.14164/j.cnki.cn11-5581/r.2017.20.065

[58] Zhou HQ Zeng G. Application of S. boulardii in infantile rotavirus enteritis. China Med Pharm. (2017) 7(20):54–6.

[59] Cao YD Wen Z. Effect of Saccharomyces boulardii sachets on inflammatory reaction and safety in children with acute diarrhea. China Pharm. (2018) 27(17):78–80. 10.3969/j.issn.1006-4931.2018.17.024

[60] Chen SH. Efficacy of Saccharomyces boulardii in the treatment of infantile acute diarrhea and its influence on cellular immune function. Chin J Proctol. (2018) 38(11):41.

[61] Fan XL. Efficacy observation of S. boulardii in the treatment of pediatric rotavirus enteritis. Integrated Med Imag Res Med Appl. (2018) 2(12):255–6.

[62] Jin L Shen ML. Clinical observation of S. boulardii in the treatment of children with acute diarrhea. Chin J Prim Med Pharm. (2018) 25(2):161–3. 10.3760/cma.j.issn.1008-6706.2018.02.007

[63] Li WJ. Efficiency of Saccharomyces boulardii sachets combined with diosmectite powder in treatment of children with viral diarrhea. J Clin Med Pract. (2018) 22(19):85–7. 10.7619/jcmp.201819024

[64] Sun B. Observation on the clinical effect of S. boulardii combined with diosmectite powder in the treatment of rotavirus enteritis in children. Chin J People’s Health. (2018) 30:43–5. 10.3969/j.issn.1672-0369.2018.23.021

[65] Sun Q. Clinical efficacy analysis of S. boulardii powder in the treatment of acute pediatric diarrhea. J Bethune Med Sci. (2018) 16(1):82–3. 10.16485/j.issn.2095-7858.2018.01.041

[66] Tang QL. Effect of S. boulardii on cellular immune function in children with diarrhea. Shenzhen J Integrated Trad Chin Western Med. (2018) 28(7):162–3. 10.16458/j.cnki.1007-0893.2018.07.078

[67] Wang H Yao L. Analysis of the clinical efficacy of S. boulardii in the treatment of pediatric acute diarrhea. Henan Med Res. (2018) 27:1645–6. 10.3969/j.issn.1004.437X.2018.09.061

[68] Weng DZ. Efficacy analysis of S. boulardii in the adjuvant treatment of pediatric rotavirus enteritis. Mod Diag Treat. (2018) 5:736–7.

[69] Yang P. Analysis of the efficacy of S. boulardii in the treatment of pediatric acute diarrhea. World Latest Med Inform. (2018) 18(31):90. 10.19613/j.cnki.1671-3141.2018.31.067

[70] Yao HY. Efficacy of S. boulardii sachets combined with diosmectite powder in treating acute pediatric diarrhea and its value in reducing diarrhea control time and total diarrhea duration. Capital Food Med. (2018) 27(1):27.

[71] Zhang H Deng GX Wang Y. The impact of S. boulardii on inflammatory responses in children with rotavirus enteritis. Shenzhen J Integrated Trad Chin Western Med. (2018) 28(5):103–5. 10.16458/j.cnki.1007-0893.2018.05.048

[72] Zhao L. Study on the application value of Saccharomyces boulardii in the treatment of pediatric diarrhea. China Mod Doctor. (2018) 56(36):57–60.

[73] Gao Z Liu F. The efficacy of S. boulardii and SiLianKang in the treatment of rotavirus diarrhea and their impact on myocardial enzymes. Shanxi Med J. (2019) 48(1):77–80. 10.3969/j.issn.0253-9926.2019.01.030]

[74] Gu HM. Analysis of the clinical value of combined treatment with Saccharomyces boulardii powder and smectite in pediatric diarrhea. Basic Med Forum. (2019) 23(5):646–7. 10.19435/j.1672-1721.2019.05.036

[75] Li J Zhang S Liu J Li Y. Observation of the curative effect of Saccharomyces boulardii powder combined with montmorillonite suspension in the treatment of children with rotavirus enteritis. J Chin Physician. (2019) 21(1):119–22. 10.3760/cma.j.issn.1008-1372.2019.01.032

[76] Liang XM. Analysis of the efficacy of Saccharomyces boulardii in the treatment of pediatric acute diarrhea and its impact on cellular immune function. CAP Food Med. (2019) 10:63–4.

[77] Wang XG Hou L. Effect of diosmectite powder combined with Saccharomyces boulardii on acute gastroenteritis in children and its influence on serum inflammatory factors. J Hunan Norm Univ (Medical Sci). (2019) 6(5):113–6.

[78] Fu W Wei YD. The impact of combined treatment with smectite and Saccharomyces boulardii on inflammatory responses and symptom improvement in children with acute gastroenteritis. Shanxi Med J. (2020) 49(9):1141–2. 10.3969/j.issn.0253-9926.2020.09.031

[79] Lu P. Observation on the efficacy of Saccharomyces boulardii powder in the treatment of pediatric rotavirus enteritis. Chin Med J Metall Indus. (2020) 37(2):241–2.

[80] Chen DD Yin YY Gui DM. Effects of Saccharomyces boulardii on symptom improvement and serum inflammatory factors in children with acute infectious diarrhea. Hebei Med. (2021) 27(2):330–4. 10.3969/j.issn.1006-6233.2021.02.033

[81] Gao HY. Efficacy evaluation of Saccharomyces boulardii powder combined with smectite powder in treating children with acute diarrhea. Pract Integrat Trad Chin Western Med in Clin Pract. (2021) 21(19):130–1. 10.13638/j.issn.1671-4040.2021.19.063

[82] Ji WF. Effect of Saccharomyces boulardii on acute diarrhea in children and its influence on T lymphocytes and gastrointestinal hormones. Matern Child Health Care of China. (2021) 36(21):4970–2.

[83] Li M. Clinical effect analysis of Saccharomyces boulardii powder in treating children with acute diarrhea. Smart Healthcare. (2021) 7(3):161–3. 10.19335/j.cnki.2096-1219.2021.3.054

[84] Xiu WS. Clinical efficacy of Saccharomyces boulardii in the treatment of pediatric rotavirus gastroenteritis. Chin J Mod Drug Appl. (2021) 15(11):186–8. 10.14164/j.cnki.cn11-5581/r.2021.11.068

[85] Fan YL Li MY Li XF. Analysis of the efficacy of Saccharomyces boulardii combined with oral rehydration salts in the treatment of pediatric rotavirus enteritis. Shanxi Med J. (2022) 51(22):2568–70. 10.3969/j.issn.0253-9926.2022.22.012

[86] Fu HB. Effect of probiotics combined with diosmectite powder on infantile diarrhea and its influence on gastrointestinal hormones and inflammatory factors. Chin J Coal Industry Med. (2022) 25(6):654–8. 10.11723/mtgyyx1007-9564202206021

[87] Liu C. The effect of Bula’s [Saccharomyces boulardii] powder combined with diosmectite powder on the levels of TNF-α, IL-10 and IL-13 in children with rotavirus infectious diarrhea. Smart Healthcare. (2022) 8(13):102–4. 10.19335/j.cnki.2096-1219.2022.13.032

[88] Xu XY Wang SJ. Effect of Saccharomyces boulardii sachets combined with diosmectite powder on rotavirus infectious diarrhea in children and its influence on immune function. Clin Med Res Practice. (2022) 7(18):80–2. 10.19347/j.cnki.2096-1413.202218022

[89] Zhai Y. The effect of Saccharomyces boulardii in adjuvant therapy for infantile rotavirus enteritis and its influence on intestinal flora. Chin J Coal Industry Med. (2022) 42(2):6–8.

[90] Cao RM. Study on the impact of S. boulardii on T lymphocytes and gastrointestinal hormones in children with acute diarrhea. Women’s Health Res. (2023) 10:47–67.

[91] Li YW Kuang HL Pan X Zhang J. The efficacy and safety of S. boulardii powder combined with diosmectite powder in the treatment of acute diarrhea in children. Guide of China Med. (2023) 21(35):21–4. 10.15912/j.cnki.gocm.2023.35.013

[92] Wang YY. Prospective study on the impact of S. boulardii on gut flora and immune function in children with rotavirus enteritis. J Jilin Med. (2023) 44(4):1042–5.

[93] Chen YC. Application of S. boulardii powder combined with diosmectite powder in children with diarrhea. Pract Clinic J Integr Tradition Chin Western Med. (2024) 24(3):68–70. 10.13638/j.issn.1671-4040.2024.03.020

[94] Szajewska H Dziechciarz P Mrukowicz J. Meta-analysis: smectite in the treatment of acute infectious diarrhoea in children. Aliment Pharm Thera. (2006) 23(2):217–27. 10.1111/j.1365-2036.2006.02760.x 16393300

[95] Pérez-Gaxiola G Cuello-García CA Florez ID Pérez-Pico VM. Smectite for acute infectious diarrhoea in children. Cochrane Database Syst Rev. (2018) 4(4):CD011526. 10.1002/14651858.CD011526.pub2 29693719 PMC6494641

[96] McFarland LV Srinivasan R Setty RP Ganapathy S Bavdekar A Mitra M Specific probiotics for the treatment of pediatric acute gastroenteritis in India: a systematic review and meta-analysis. JPGN Reports. (2021) 2(3):e079. 10.1097/PG9.0000000000000079 37205949 PMC10191489

[97] Li Z Zhu G Li C Lai H Liu X Zhang L. Which probiotic is the most effective for treating acute diarrhea in children? A Bayesian network meta-analysis of randomized controlled trials. Nutrients. (2021) 13(12):4319. 10.3390/nu13124319 34959871 PMC8706888

[98] Huang R Xing HY Liu HJ Chen ZF Tang BB. Efficacy of probiotics in the treatment of acute diarrhea in children: a systematic review and meta-analysis of clinical trials. Translational Pediatr. (2021) 10(12):3248–60. 10.21037/tp-21-511 PMC8753473 35070839

[99] Fu HB Li J Xu X Xia C Pan Y. Effectiveness and safety of S. boulardii for the treatment of acute gastroenteritis in the pediatric population: a systematic review and meta-analysis of randomized controlled trials. Comput Math Methods Med. (2022) 2022:1–10. 10.1155/2022/6234858 PMC9514928 36176742

[100] Minaz A Alam R Jiwani U Vadsaria K Khan A Ishaq A Efficacy of probiotics for treatment of acute or persistent diarrhoea in children from birth until 10 years: systematic review and meta-analysis. J Global Health. (2024) ).14:04236. 10.7189/jogh.14.04236 PMC11659791 39703988

[101] Eren M Dinleyici EC Vandenplas Y. Clinical efficacy comparison of Saccharomyces boulardii and yogurt fluid in acute non-bloody diarrhea in children: a randomized, controlled, open label study. Am J Trop Med Hyg. (2010) 82(3):488–91. 10.4269/ajtmh.2010.09-0529 20207879 PMC2829915

[102] Duan W Zhou C Luo M Zuo X. Effects of Saccharomyces boulardii powder on disease progression of rotavirus gastroenteritis in children. Mod Dig Interv. (2017) 22(5):692–4. 10.3969/j.issn.1672-2159.2017.05.028

[103] Zhao YF Shao XT Xu B Zhou X Lu Q. Influence of Saccharomyces boulardii on expression of serum IL-6 and TNF-α of children with rotavirus infections. Chin J Nosocomiol. (2017) 27(21):4989–91. 10.11816/cn.ni.2017-170946

[104] Altcheh J Carosella MV Ceballos A Andreas UD Jofre SM Marotta C Randomized, direct comparison study of Saccharomyces boulardii CNCM I-745 versus multi-strained Bacillus clausii probiotics for the treatment of pediatric acute gastroenteritis. Medicine (Baltimore). (2022) 101(36):e30500. 10.1097/MD.0000000000030500 36086703 PMC9646502

[105] Wei Q Song LY Rao R Yang HW Wen YP Lv L The impact of combined therapy with Lactobacillus acidophilus and montmorillonite powder on the inflammatory response in pediatric rotavirus enteritis. Interl Arch Aller Immunol. (2025) 186(7):689–95. 10.1159/000542593 39586286

[106] Pieścik-Lech M Urbańska M Szajewska H. Lactobacillus GG (LGG) and smectite versus LGG alone for acute gastroenteritis: a double-blind, randomized controlled trial. Eur J Pediatr. (2013) 172(2):247–53. 10.1007/s00431-012-1878-2 23114849 PMC3560958

[107] Cai R Deng Q. Clinical effect of Saccharomyces boulardii combined with montmorillonite powder in the treatment of infantile rotavirus diarrhea. Chin J Clin Rational Drug Use. (2025) 18(33):23–5.

[108] Zhou B. Clinical effect observation of Saccharomyces boulardii sachets combined with montmorillonite powder in rotavirus enteritis. Medical Forum. (2025) 29(24):68–70. 10.19435/j.1672-1721.2025.24.021

