# Supplementary material for: Saccharomyces boulardii CNCM I-745 and smectite treatment for pediatric acute gastroenteritis in China: a systematic review and meta-analysis
Source: Front Pediatr. 2026 Feb 19;14:1747695. doi: 10.3389/fped.2026.1747695 (PMC12961810; doi:10.3389/fped.2026.1747695)
Supplement: Supplementary file 1 [file Supplementaryfile1.docx]

**Supplementary Data**

Supplementary Figure S1. Funnel plot of publication bias: cured

Supplementary Figure S2. Risk of Bias summary figure.

Supplementary Figure S3. Forest plot of duration of PAGE by country

Supplementary Table S1. PRISMA Checklist

Supplementary Table S2. Search Strategies

Supplementary Table S3. Examples of excluded trials

Supplementary Table S4. Study population characteristics and safety data by intervention groups

Supplementary Table S5. Primary Outcomes

Supplementary Table S6. Sub-group analysis of primary outcomes

Supplementary Table S7. Secondary outcomes

Supplementary Table S8. Inflammatory marker outcomes

Supplementary Table S9. GRADE assessment


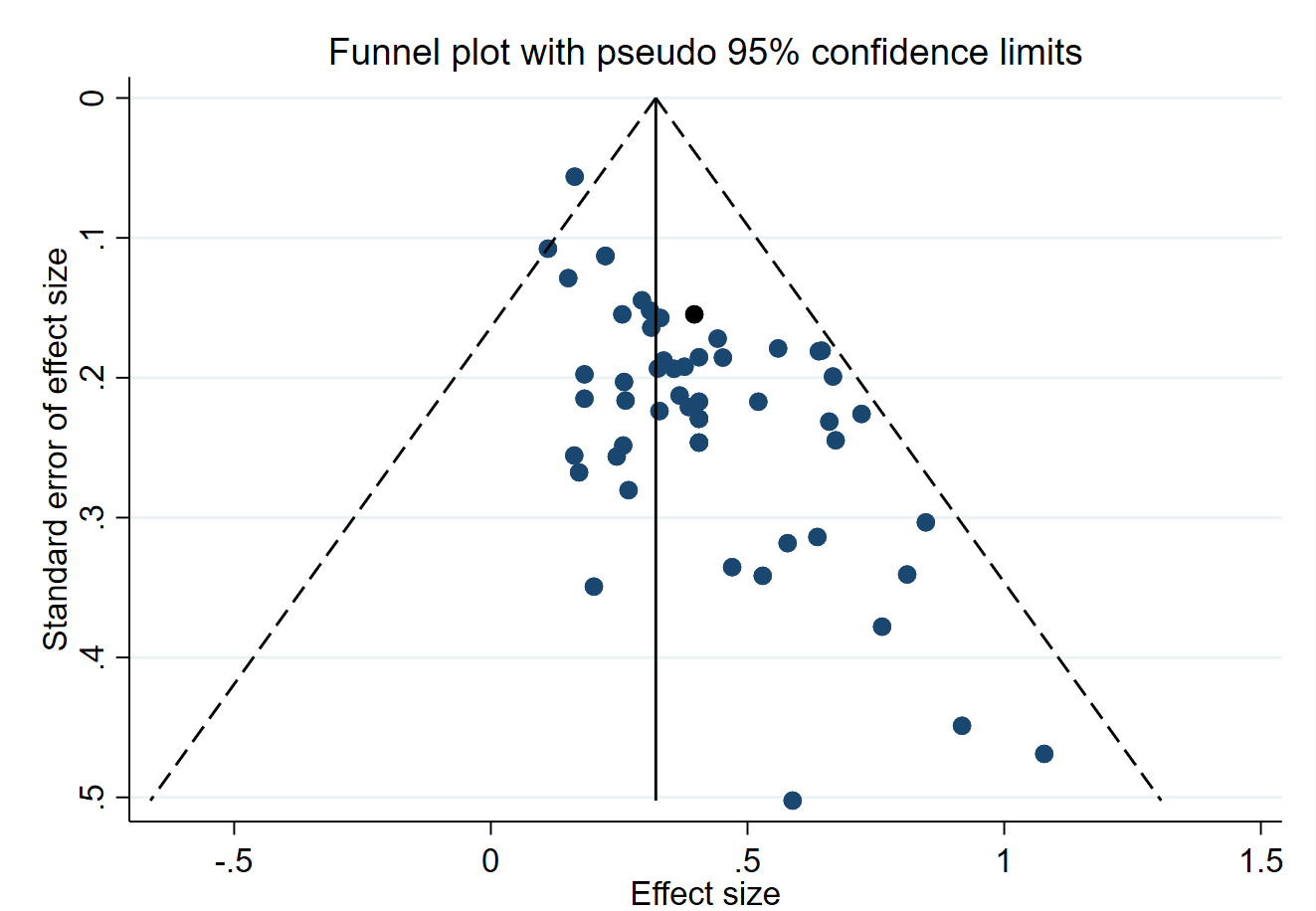


**Supplementary Figure S1**. Funnel plot of publication bias: cured

**Supplementary Figure S2.** Risk of Bias summary figure.


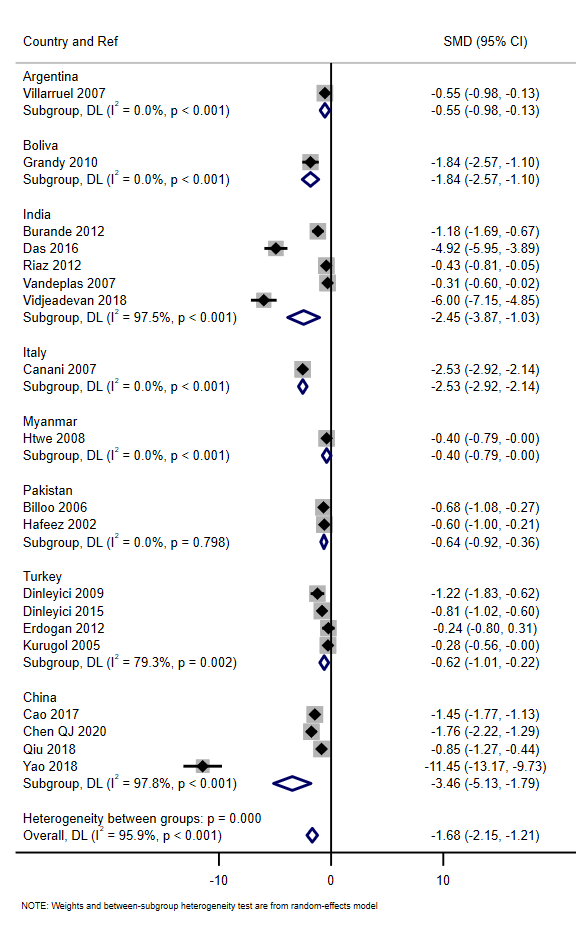


**Supplementary Figure S3**. Forest plot of duration of PAGE by country comparing *S. boulardii* CNCM I-745 to controls. Modified from Szajewska et al. 2020 and McFarland and Li 2025.

**Supplementary Table S1.** PRISMA Checklist

| **Section and Topic** | **Item #** | **Checklist item** | **Location where item is reported (section/Fig/Table)** |
| --- | --- | --- | --- |
| **TITLE** | | |  |
| Title | 1 | Identify the report as a systematic review. | title page |
| **ABSTRACT** | | |  |
| Abstract | 2 | See the PRISMA 2020 for Abstracts checklist. | abstract |
| **INTRODUCTION** | | |  |
| Rationale | 3 | Describe the rationale for the review in the context of existing knowledge. | 1. Introduction |
| Objectives | 4 | Provide an explicit statement of the objective(s) or question(s) the review addresses. | 1. Introduction |
| **METHODS** | | |  |
| Eligibility criteria | 5 | Specify the inclusion and exclusion criteria for the review and how studies were grouped for the syntheses. | 2.2 |
| Information sources | 6 | Specify all databases, registers, websites, organisations, reference lists and other sources searched or consulted to identify studies. Specify the date when each source was last searched or consulted. | 2.1 |
| Search strategy | 7 | Present the full search strategies for all databases, registers and websites, including any filters and limits used. | Suppl Table S2 |
| Selection process | 8 | Specify the methods used to decide whether a study met the inclusion criteria of the review, including how many reviewers screened each record and each report retrieved, whether they worked independently, and if applicable, details of automation tools used in the process. | 2.3 |
| Data collection process | 9 | Specify the methods used to collect data from reports, including how many reviewers collected data from each report, whether they worked independently, any processes for obtaining or confirming data from study investigators, and if applicable, details of automation tools used in the process. | 2.3 |
| Data items | 10a | List and define all outcomes for which data were sought. Specify whether all results that were compatible with each outcome domain in each study were sought (e.g. for all measures, time points, analyses), and if not, the methods used to decide which results to collect. | 2.5 and 2.6 |
|  | 10b | List and define all other variables for which data were sought (e.g. participant and intervention characteristics, funding sources). Describe any assumptions made about any missing or unclear information. | 2.3 |
| Study risk of bias assessment | 11 | Specify the methods used to assess risk of bias in the included studies, including details of the tool(s) used, how many reviewers assessed each study and whether they worked independently, and if applicable, details of automation tools used in the process. | 2.4 |
| Effect measures | 12 | Specify for each outcome the effect measure(s) (e.g. risk ratio, mean difference) used in the synthesis or presentation of results. | 2.8 |
| Synthesis methods | 13a | Describe the processes used to decide which studies were eligible for each synthesis (e.g. tabulating the study intervention characteristics and comparing against the planned groups for each synthesis (item #5)). | 2.2 |
|  | 13b | Describe any methods required to prepare the data for presentation or synthesis, such as handling of missing summary statistics, or data conversions. | 2.8 |
|  | 13c | Describe any methods used to tabulate or visually display results of individual studies and syntheses. | 2.8 |
|  | 13d | Describe any methods used to synthesize results and provide a rationale for the choice(s). If meta-analysis was performed, describe the model(s), method(s) to identify the presence and extent of statistical heterogeneity, and software package(s) used. | 2.8 |
|  | 13e | Describe any methods used to explore possible causes of heterogeneity among study results (e.g. subgroup analysis, meta-regression). | 2.7 |
|  | 13f | Describe any sensitivity analyses conducted to assess robustness of the synthesized results. | 2.8 |
| Reporting bias assessment | 14 | Describe any methods used to assess risk of bias due to missing results in a synthesis (arising from reporting biases). | 2.8 |
| Certainty assessment | 15 | Describe any methods used to assess certainty (or confidence) in the body of evidence for an outcome. | 2.4, 2.8 |
| **RESULTS** | | |  |
| Study selection | 16a | Describe the results of the search and selection process, from the number of records identified in the search to the number of studies included in the review, ideally using a flow diagram. | Figure 1 3.1 |
|  | 16b | Cite studies that might appear to meet the inclusion criteria, but which were excluded, and explain why they were excluded. | Table S3 3.1 |
| Study characteristics | 17 | Cite each included study and present its characteristics. | Table S4,  3.2 |
| Risk of bias in studies | 18 | Present assessments of risk of bias for each included study. | 3.3 |
| Results of individual studies | 19 | For all outcomes, present, for each study: (a) summary statistics for each group (where appropriate) and (b) an effect estimate and its precision (e.g. confidence/credible interval), ideally using structured tables or plots. | Fig 2-5 Table 1 |
| Results of syntheses | 20a | For each synthesis, briefly summarise the characteristics and risk of bias among contributing studies. | Fig. S2 3.3 |
|  | 20b | Present results of all statistical syntheses conducted. If meta-analysis was done, present for each the summary estimate and its precision (e.g. confidence/credible interval) and measures of statistical heterogeneity. If comparing groups, describe the direction of the effect. | Fig 2-5 Table 1 3.5-3.8 |
|  | 20c | Present results of all investigations of possible causes of heterogeneity among study results. | Table S6 3.5-3.7 |
|  | 20d | Present results of all sensitivity analyses conducted to assess the robustness of the synthesized results. | 3.6 |
| Reporting biases | 21 | Present assessments of risk of bias due to missing results (arising from reporting biases) for each synthesis assessed. | nr |
| Certainty of evidence | 22 | Present assessments of certainty (or confidence) in the body of evidence for each outcome assessed. | Table 1 Suppl Table S9 |
| **DISCUSSION** | | |  |
| Discussion | 23a | Provide a general interpretation of the results in the context of other evidence. | 4.0 |
|  | 23b | Discuss any limitations of the evidence included in the review. | 4.0 |
|  | 23c | Discuss any limitations of the review processes used. | 4.0 |
|  | 23d | Discuss implications of the results for practice, policy, and future research. | 4.0 |
| **OTHER INFORMATION** | | |  |
| Registration and protocol | 24a | Provide registration information for the review, including register name and registration number, or state that the review was not registered. | 2.0 |
|  | 24b | Indicate where the review protocol can be accessed, or state that a protocol was not prepared. | 2.0 |
|  | 24c | Describe and explain any amendments to information provided at registration or in the protocol. | na |
| Support | 25 | Describe sources of financial or non-financial support for the review, and the role of the funders or sponsors in the review. | title page |
| Competing interests | 26 | Declare any competing interests of review authors. | title page |
| Availability of data, code and other materials | 27 | Report which of the following are publicly available and where they can be found: template data collection forms; data extracted from included studies; data used for all analyses; analytic code; any other materials used in the review. | Supplementary data files |

*From:*  Page MJ, McKenzie JE, Bossuyt PM, Boutron I, Hoffmann TC, Mulrow CD, et al. The PRISMA 2020 statement: an updated guideline for reporting systematic reviews. BMJ 2021;372:n71. doi: 10.1136/bmj.n71

**Supplementary Table S2**. Search strategies

| **Literature Database** | **Search strategy and keywords** |
| --- | --- |
| China National Knowledge Infrastructure | Search strategy  (TKA % '布拉氏酵母菌' +'布拉酵母菌' +'亿活' ) and (TKA % '腹泻' + '急性腹泻' + '轮状病毒性肠炎'+ '秋季腹泻'-'抗生素相关'-'幽门')  and (TKA % '小儿' + '儿童' + '婴幼儿'+ '新生儿'+ '患儿')  Keywords  布拉氏酵母菌、布拉酵母菌、亿活，腹泻、小儿急性腹泻、小儿腹泻、婴幼儿腹泻、新生儿腹泻、新生儿轮状病毒性肠炎、秋季腹泻、儿童腹泻、腹泻患儿  ["saccharomyces boulardii","Yihuo","diarrhea", "acute diarrhea", "rotavirus enteritis", "autumn diarrhea", "acute diarrhea in children", "infantile diarrhea","children diarrhea", "neonatal diarrhea", "diarrhea infants"] |
| China Biology Medicine disc | Search strategy #1"布拉氏酵母菌"[常用字段] OR "布拉酵母菌"[常用字段] OR "亿活"[常用字段] #2"腹泻"[常用字段] OR "急性腹泻"[常用字段] OR "轮状病毒性肠炎"[常用字段] OR "秋季腹泻"[常用字段] NOT "抗生素相关"[常用字段] NOT "幽门"[常用字段] #3"小儿"[常用字段] OR "儿童"[常用字段] OR "新生儿"[常用字段] OR "婴幼儿"[常用字段] OR "患儿"[常用字段] (#1) AND (#2) AND (#3) [translation: #1 “Saccharomyces boulardii" [common field] OR "Saccharomyces boulardii" [common field] OR "Billion Live" [common field] #2 "Diarrhea" [Common Field] OR "Acute Diarrhea" [Common Field] OR "Rotavirus Enteritis" [Common Field] OR "Autumn Diarrhea" [Common Field] NOT "Antibiotic Related" [Common Field] NOT "Pylorus" [Common Field] #3 "Child" [Frequently Used Field] OR "Child" [Frequently Used Field] OR "Newborn" [Frequently Used Field] OR "Infant" [Frequently Used Field] OR "Sick Child" [Frequently Used Field]  Keywords (same as above) but different strategy |
| Embase | ('pediatrics'/exp OR 'pediatrics') AND ('acute diarrhea'/exp OR 'acute diarrhea') AND ('China'/exp OR 'China') AND 'controlled trial' AND ‘Saccharomyces boulardii’ |
| Google Scholar | clinical trials AND acute pediatric diarrhea OR rotavirus AND Saccharomyces boulardii AND China NOT animals AND smectite |
| PubMed | (“probiotics” [MeSH Terms] OR “probiotics” [All Fields]) AND [“pediatric” AND/OR “acute diarrhea” [MeSH Terms] AND “clinical trials” [All Fields] AND “China” [All Fields] AND “Saccharomyces boulardii” [All Fields] AND "smectite"). |

**Supplementary Table S3.** Excluded trials (n=10) of PAGE trials with smectite

| **Reference** | **Types of Probiotics compared** | **Reason excluded** |
| --- | --- | --- |
| Feng N 2018 | *S. boulardii* vs mezlocillin | Retrospective study |
| Huang K 2018 | *S. boulardii* vs open control | All received adjunctive treatments (potassium and zinc) |
| Li Gui-nan 2014 | *S. boulardii* vs open control | Prevention of PAGE not treatment |
| Liu T 2020 | *S. boulardii* vs "Bifido Quad" | Direct comparison of two probiotics, no non-probiotic control |
| Liu ZY 2019 | *S. boulardii* vs open control | All received potassium as adjunctive treatment |
| Shi F 2015 | *S. boulardii* vs open control | smectite only in control group |
| Wang Dan 2020 | *S. boulardii* vs open control | Unclear if randomized |
| Wang WH 2016 | *S. boulardii* vs open control | Controls given antibiotics, S.b. did not |
| Zhang M 2014 | *S. boulardii* vs open control | no smectite documented in either group |
| Zhang XQ 2014 | *S. boulardii* vs open control | smectite only in control group |
| Zou J 2018 | *S. boulardii* vs open control | chronic diarrhea |

**References:**

Feng N, Lei Z, Yang H, Hu L. Clinical efficacy of *Saccharomyces boulardii* combined with mezlocillin in the treatment of children with infectious diarrhea and effect on serum CRP,PCT and IL-8. *Chinese J Integr Tradit West Med Dig*. 2018;26(2):194-197.

Huang Kai. Efficacy of *Saccharomyces boulardii* in the treatment of pediatric acute diarrhea and its impact on cellular immune function. *North Pharmaceut J.* 2018, 15(4): 147-161.

Liu T. Analysis of the clinical effect of treating children with rotavirus gastroenteritis with *Saccharomyces* *boulardii* and *Bifidobacterium* quadruple live bacteria. *Cardiovasc Dis Integr Tradit Chinese West Med*. 2020;8(34):57-63. [doi:10.16282/j.cnki.cn11-9336/r.2020.34.040]

Liu Zhaoyun. Analysis of the Efficacy of *Saccharomyces boulardii* powder in the treatment of pediatric acute diarrhea. *J Med Theor & Prac*. 2019, 32(21): 3512-3513. [doi: 10.19381/j. issn.1001－7585.2019.21.059]

Shi Fan. Analysis of the Efficacy of *S. boulardii* in treating acute diarrhea in children. *Jilin Medical J.* 2015;36(11): 9522.

Wang Dan, Cheng Yiwu, Zhou Haiqun. Effects of *Saccharomyces boulardii* combined with montmorillonite powder on acute diarrhea in children and T-cell subsets. *Drug Eval Res*. 2020;43(6):1095-1098. [doi: 10.7501/j.issn.1674-6376.2020.06.020]

Wang Weihong. Cinical research on the prevention and treatment of acute diarrhea in children with *Saccharomyces boulardii*. *J Prev Med Chinese People’s Lib Army*. 2016;34(4):302. [doi:10.13704/j.cnki.jyyx.2016.s2.273]

Zhang Min, Chen Hui. Clinical Efficacy of *Saccharomyces boulardii* in the treatment of infantile rotavirus enteritis. *Practical Medicine J.* 2014;30(22): 3698-3699. [doi: 10.3969/j.issn.1006-5725.2014.22.059]

Zhang Xiao-qing, Hu Wen-hui. Effect of *Saccharomyces boulardii* on serum cytokines and clinical efficacy in infantile rotavirus enteritis. *Chinese Remedies & Clinics*. 2014;14(1): 72-73. [doi: 10.11655/zgywylc.2014.01.031]

Zou Jiaoyun. Efficacy of montmorillonite powder combined with *Saccharomyces boulardii* in the treatment of pediatric acute diarrhea. *Practical Clinical Medicine.* 2018, (19):63-64. [doi: 10.13764/j.cnki.lcsy.2018.03.023]

**Supplementary Table S4.** Study population characteristics

| **Reference** | **N enrolled** | **Age range** | **Diarrhea prior to enrollment (days)** | **Etiology of diarrhea** | **Adjunctive treatments*** | ***S. boulardii* dose (mg/d)** | **Duration study treatment (days)** | **Adverse event ^a^** |
| --- | --- | --- | --- | --- | --- | --- | --- | --- |
| Hu XY 2002 | 103 | 3 mon-3 yr | 2 | rota+ | Rehy, diet, ribavirin | <1 yr: 250 1-3 yrs: 375 | 3 | nr |
| Ji DZ 2009 | 100 | 2 mon- 7 yr | <3 | nr | Rehy, diet, abx prn | <1 yr: 250 >1 yr: 500 | 7-14 | state |
| Wei WQ 2011 | 105 | 5 mon-2.5 yr | < 3 | rota+ | Rehy, ribavirin | <1 yr: 250 >1 yr: 500 | 5 | state |
| Xiao QZ 2011 | 69 | <3 yr | nr | rota+ | MMT, Rehy | <1 yr: 250 1-3 yrs: 500 | nr | state |
| Zhang JB 2011 | 92 | 2 mon-7 yr | < 3 | nr | Rehy, abx prn | <1 yr: 250 >1 yr: 500 | 7-14 | nr |
| Fu SL 2012 | 100 | 1 mon-8 yr | < 2 | nr | Rehy, diet | 250 | 5 | nr |
| Wang DE 2013 | 92 | 5 d-28 d neonates | nr | rota+ | Rehy | 250 | 3-5 | state |
| Zhang ZJ 2013 | 92 | <1.6 yr | < 4 | nr | Rehy, abx prn | 250 | ~6 (los) | state |
| Xiong B 2014 | 92 | 6 mon-3 yr | < 3 | rota+ | Rehy | < 1 yr: 250 >1 yr: 500 | 7-14 (los) | state |
| Yuan YH 2014 | 76 | <1.3 yr | < 2 | rota+ | Rehy, antivirals | <1 yr: 125 >1 yr: 500 | 3 | state |
| Ding L 2015 | 98 | 1 mon-3 yr | < 2 | rota+ | MMT, Rehy | <1 yr: 250 >1 yr: 500 | 7 (los) | state |
| Wu L 2015 | 127 ^b^ | 2 mon-8 yr | < 2 | rota+ | Rehy | <3 yrs: 250 > 3 yrs: 500 | 5 | state |
| Xu ZX 2015 | 53 | 1 mon-5 yr | < 6 | nr | Rehy anti-inflam | < 3yr: 250 >3 yrs: 500 | 5 | state |
| Sun XL 2016 | 164 | 4 mon-3 yr | < 2 | nr | Rehy, abx prn | <1 yr: 250 >1 yr: 500 | 7 | nr |
| Xia L 2016 | 128 | 3 mon-2 yr | < 2 | rota+ | Rehy | <1 yr: 250 > 1 yr: 500 | nr | state |
| Yang NF 2016 | 68 | <6 yr | 5 | nr | Rehy | < 3 yr: 250 > 3 yr: 500 | 6 | mild AE, no data |
| Zhao JF 2016 | 88 | 3 mon- 3.5 yr | < 2 | nr | Rehy, antivirals | < 1 yr: 250 > 1 yr: 500 | 4-7 | state |
| Shi HJ 2017 | 60 | 1 mon- 8 yr | < 7 | nr | Rehy | < 3 yr: 250 > 3 yrs: 500 | 6 | nr |
| Yuan S 2017 | 100 | <6 yrs | < 2 | nr | Rehy, abx prn | <1 yr: 250 > 1 yr: 500 | nr | state |
| Zhang J 2017 | 100 | 2 yr- 8 yr | nr | nr | Rehy | < 3 yr: 250 > 3 yr: 500 | 6 | nr |
| Zhong S 2017 | 60 | 2 yr- 9 yr | nr | nr |  | 500 | 3 | nr |
| Zhou HQ 2017 | 150 | 1 mon- 1 yr | < 3 | rota+ | Rehy | 250 | 3 | nr |
| Cao Y 2018 | 120 | 6 mon-2 yr | < 3 | nr | Rehy, anti-inflam prn | <3 yrs: 250 > 3 yrs: 500 | 6 | state |
| Chen SH 2018 | 92 | 1 yr- 6 yr | < 10 | nr | Rehy, antiinf prn | < 3 yr: 250 > 3 yr: 500 | nr | nr |
| Fan XL 2018 | 84 | 7 mon- 4 yr | < 6 | rota+ | Rehy | < 3 yr: 250 > 3 yr: 500 | nr | state |
| Jin L 2018 | 100 | 2 mon- 7 yr | nr | nr | Rehy | < 3 yr: 250 > 3 yr: 500 | 7 | nr |
| Li W 2018 | 120 | 6 mon-6 yr | < 2 | Viral, mixed | Rehy, diet | 250 | 5 | nr |
| Sun BJ 2018 | 94 | 3 mon- 3 yr | < 5 | rota+ | Rehy, antivirals | < 1 yr: 125 > 1 yr: 500 | 12 weeks | 4% vs 8.5% |
| Sun Q 2018 | 96 | 5 mon- 5 yr | nr | nr | Rehy | <1 yr: 250 > 1 yr: 500 | 7 | nr |
| Tang QL 2018 | 86 | 5 mon- 5 yr | < 14 | nr |  | 250 | 3 | nr |
| Wang HN 2018 | 92 | 1 yr- 6 yr | < 1.3 | nr | diet, abx prn | < 1 yr: 250 >1 yr: 500 | 7 | nr |
| Weng DZ 2018 | 82 | 6 mon- 3 yr | nr | rota+ | MMT, Rehy | < 3 yr: 250 > 3 yr: 500 | nr [los] | nr |
| Yang Ping 2018 | 82 | 3.7 mon- 3 yr | < 3 | nr | Rehy, antivirals prn | < 3 yr: 250 > 3 yr: 500 | nr | nr |
| Yao HY 2018 | 112 | 1 yr- 10 yr | < 2 | nr |  | 250 | 6 | nr |
| Zhang H 2018 | 150 | 3 mon- 3 yr | < 4 | rota+ | Rehy | <1 yr: 250 >1 yr: 500 | 7 | nr |
| Zhao L 2018 | 80 | 1 mon- 8 yr | < 10 | nr | Rehy | < 3 yr: 250 > 3 yr: 500 | 5 | 22% vs 20% |
| Gao ZH 2019 | 188 ^b^ | 3 mon- 6 yr | < 2 | rota+ | Rehy | < 3 yr: 250 > 3 yr: 500 | 3 | nr |
| Gu HM 2019 | 70 | < 6 yr | **nr** | bacterial^c^ | Rehy, diet, anti-inflam | < 3 yr: 250 > 3 yr: 500 | 6 | nr |
| Li Juan 2019 | 114 | 6 mon-3 yr | < 3 | rota+ | MMT, Rehy, antiviral prn | <1 yr: 125 > 1 yr: 500 | 3 | 8.8% vs 5.2% |
| Liang XM 2019 | 88 | 1 yr- 9 yr | < 2 | nr | Rehy | <1 yr: 250 > 1 yr: 500 | nr | nr |
| Wang X 2019 | 246 | 5 mon-3 yr | < 2.1 | nr | Rehy | <1 yr: 250 > 1 yr: 500 | 7 | some AEs, no data by group |
| Fu W 2020 | 102 | 6 mon- 1.5 yr | < 4 | rota+ | Rehy | <1 yr: 250 > 1 yr: 500 | 7 | 3.9% vs 7.8% |
| Lu P 2020 | 88 | <2 yr | < 4 | rota+ | Rehy | <1 yr: 250 >1 yr: 500 | 7 | nr |
| Chen D 2021 | 160 | 4 mon-5 yr | < 1.3 | nr | Rehy, AM prn | <3 yrs: 250 > 3 yrs: 500 | 7 | 2.5% vs 6.2% |
| Gao HY 2021 | 92 | 4 mon- 6 yr | < 2 | nr | Rehy, AM prn | < 3 yr: 250 > 3 yr: 500 | 7 | nr |
| Ji WF 2021 | 130 | 6 mon- 1.5 yr | < 3 | nr | Rehy, antivirals | < 1 yr: 250 > 1 yr: 500 | 3 | 4.6% vs 9.2% |
| Li M 2021 | 84 | 5 mon- 4 yr | < 1.2 | nr | Rehy | < 1 yr: 250 > 1 yr: 500 | 7 | nr |
| Xiu WS 2021 | 50 | 1 yr- 3 yr | < 3 | rota+ | Rehy | 250-1000^d^ | 7 | nr |
| Fan YL 2022 | 80 | <5 yr | < 3 | rota+ | Rehy | < 1 yr: 250 > 1 yr: 500 | 7 | 8% vs 15% |
| Fu HB 2022 | 120 | 2 yr- 6 yr | < 2 | mixed^e^ | Rehy, diet, antivirals | < 3 yr: 250 > 3 yr: 500 | 7 | 5% vs 3.3% |
| Liu C 2022 | 136 | 4 mon- 4 yr | < 5 | rota+ | Rehy, antivirals | < 3 yr: 250 > 3 yr: 500 | nr | 2.9% vs 4.4% |
| Xu XY 2022 | 80 | 6 mon- 6 yr | < 2 | rota+ | Rehy, antiinflam | < 3 yr: 250 > 3 yr: 500 | 3-5 | nr |
| Zhai YJ 2022 | 56 | < 3 yr | < 5 | rota+ | Rehy, diet, antivirals | < 1 yr: 125 > 1 yr: 250 | 7 | nr |
| Cao RM 2023 | 80 | 6 mon- 3 yr | < 3 | nr | Rehy, antivirals | < 1 yr: 250 > 1 yr: 500 | 3 | nr |
| Li YW 2023 | 124 | 2 yr- 5 yr | < 2 | nr |  | < 3 yr: 250 > 3 yr: 500 | 6 | 3.2% vs 14.5% |
| Wang YY 2023 | 62 | 6 mon- 3 yr | < 3 | rota+ | Rehy, diet, antivirals | < 1 yr: 125 > 1 yr: 500 | 7 | nr |
| Chen YC 2024 | 110 | 1 yr- 6 yr | < 8 | rota+ | Rehy, anti-inflam | < 3 yr: 250 > 3 yr: 500 | 3-5 | 1.8% vs 5.4% |

^a^ state, just a statement in the paper that no adverse reactions were noted in either group;
^b^ excludes other probiotic Bifido. strain arm (n=94) [Wu L 2015]
^c^ mixed bacterial etiologies: 60% *E. coli,* 24% *Salmonella*, 16% *Enterococcus* [Gu 2019]
^d^ Sb dose not specified by age [Xiu W 2021]
^e^ mixed etiologies: 46% bacterial, 26% viral, 28% parasitic [Fu HB 2022]

* Adjunctive treatments include diosmectite unless noted.

**Abbreviations**: abx, anti-microbials as needed; anti-inflam, anti-inflammatory; diet, diet changes; los, during length of stay in hospital; MMT, montmorillonite; mon, month; nr, not reported; Rehy, oral or intravenous rehydration; rota+, rotavirus positive diarrhea; yrs, years old

**Supplementary Table S5.** Primary Outcomes for treatment of PAGE comparing *S. boulardii* with controls

| **Reference** | **Cured in *S. boulardii:* number (%)** | **Cured in controls: number (%)** | **Duration of diarrhea in *S. boulardii:* mean days (SD)** | **Duration of diarrhea in controls: mean days (SD)** | **Total effective rate, *S. boulardii* (%)** | **Total effective rate, control (%)** |
| --- | --- | --- | --- | --- | --- | --- |
| Hu XY 2002 | 18/53 (34) | 10/50 (20) | 2.89 (0.90) | 3.34 (1.20) | 49/53 (92.5) | 39/50 (78) |
| Ji DZ 2009 | 24/46 (52.2) | 16/46 (34.8) | 5.72 (1.67) | 6.54 (1.74) | 42/46 (91.3) | 34/46 (73.9) |
| Wei WQ 2011 | 20/51 (36.4) | 11/50 (22) | 2.9 (0.9) | 3.3 (1.2) | 51/55 (92.7) | 38/50 (76) |
| Xiao QZ 2011 | 26/35 (74.3) | 15/34 (44.1) | nr | nr | 34/35 (97.1) | 27/34 (79.4) |
| Zhang JB 2011 | 40/46 (87.0) | 32/46 (69.6) | nr | nr | 45/46 (97.8) | 40/46 (87) |
| Fu SL 2012 | 37/50 (74.0) | 19/50 (38) | nr | nr | 48/50 (96) | 41/50 (82) |
| Wang DE 2013 | 33/46 (71.7) | 21/46 (45.6) | 2.9 (0.84) | 3.9 (1.0) | 45/46 (97.8) | 38/46 (82.6) |
| Zhang ZJ 2013 | 42/59 (71.2) | 12/33 (36.4) | nr | nr | 53/59 (90) | 24/33 (73) |
| Xiong B 2014 | 24/46 (52.2) | 16/46 (34.8) | 5.72 (1.67) | 6.54 (1.74) | 42/46 (91.3) | 34/46 (73.9) |
| Yuan YH 2014 | 24/38 (63.1) | 20/38 (52.6) | nr | nr | 36/39 (94.7) | 30/38 (78.9) |
| Ding L 2015 | 16/49 (32.7) | 5/45 (11.1) | 5.1 (2.1) | 6.53 (2.54) | 44/49 (83.7) | 29/45 (64.5) |
| Wu L 2015 | 36/65 (55.4) | 24/60 (40) | 3.67 (1.03) | 5.02 (1.5) | 60/65 (92.3) | 46/60 (74.2) |
| Xu ZX 2015 | 13/27 (48.1) | 5/26 (19.2) | 4.66 (1.32) | 6.31 (2.05) | 24/27 (88.9) | 17/26 (65.4) |
| Sun XL 2016 | 35/82 (43) | 27/82 (33) | 3.0 (1.1) | 4.9 (1.2) | 71/82 (87) | 57/82 (70) |
| Xia L 2016 | 42/64 (65.6) | 27/64 (42.2) | 1.94 (0.51) | 3.01 (0.87) | 63/64 (98.4) | 52/64 (81.2) |
| Yang NF 2016 | 9/34 (26.5) | 5/34 (14.7) | 4.8 (1.2) | 6.6 (1.5) | 31/34 (91.2) | 24/31 (73.5) |
| Zhao JF 2016 | 25/44 (56.8) | 18/44 (40.9) | nr | nr | 42/44 (95.5) | 33/44 (75) |
| Shi HJ 2017 | 15/30 (50) | 7/30 (23.3) | 4.69 (1.3) | 6.34 (2.06) | 28/30 (93.3) | 19/30 (63.3) |
| Yuan S 2017 | 40/50 (80) | 21/50 (42) | 3.54 (1.85) | 5.87 (2.46) | 48/50 (96) | 35/50 (70) |
| Zhang J 2017 | 27/50 (54) | 18/50 (36) | 2.73 (0.22) | 4.61 (0.32) | 46/50 (92) | 38/50 (76) |
| Zhong S 2017 | 21/30 (70) | 9/30 (30) | nr | nr | 29/30 (96.7) | 24/30 (80) |
| Zhou HQ 2017 | 73/75 (97.3) | 62/75 (82.7) | nr | nr | nr | nr |
| Cao Y 2018 | 23/60 (38.3) | 18/60 (30) | 1.23 (0.27) | 2.2 (0.38) | 56/60 (93.3) | 48/60 (80) |
| Chen SH 2018 | 19/46 (41.3) | 16/46 (34.8) | nr | nr | 43/46 (93.5) | 36/46 (78.3) |
| Fan XL 2018 | 29/42 (69) | 15/42 (35.7) | 3.8 (1.7) | 5.1 (1.6) | 38/42 (90.5) | 30/42 (71.4) |
| Jin L 2018 | 27/50 (54) | 18/50 (36) | 2.85 (1.17) | 4.3 (1.5) | 48/50 (96) | 40/50 (80) |
| Li W 2018 | 35/60 (58) | 25/60 (41.7) | 5.16 (1.01) | 6.22 (1.12) | 58/60 (96.7) | 52/60 (86.7) |
| Sun BJ 2018 | 22/47 (46.8) | 17/47 (36.2) | nr | nr | 45/47 (95.7) | 38/47 (80.8) |
| Sun Q 2018 | 26/48 (54.2) | 20/48 (41.7) | 5.6 (1.1) | 6.8 (1.3) | 46/48 (95.8) | 39/48 (81.2) |
| Tang QL 2018 | 35/43 (81.4) | 20/43 (46.5) | 3.21 (1.2) | 5.0 (1.4) | 40/43 (93) | 32/43 (74.4) |
| Wang HN 2018 | 20/46 (43.4) | 17/46 (36.9) | 2.87 (0.56) | 4.1 (0.5) | 43/46 (93.5) | 36/46 (78.3) |
| Weng DZ 2018 | 26/41 (63.4) | 18/41 (43.9) | 3.5 (1.2) | 4.7 (1.7) | 39/41 (95.1) | 33/41 (80.5) |
| Yang Ping 2018 | nr | nr | 3.08 (0.87) | 4.08 (0.9) | nr | nr |
| Yao HY 2018 | nr | nr | 2.53 (0.3) | 4.83 (0.5) | nr | nr |
| Zhang Hong 2018 | 50/75 (66.7) | 43/75 (57.3) | 1.84 (0.47) | 3.06 (0.8) | 72/75 (96) | 63/75 (84) |
| Zhao L 2018 | 32/40 (80) | 23/40 (57.5) | nr | nr | 39/40 (97.5) | 34/40 (85) |
| Gao ZH 2019 | 55/94 (59) | 37/94 (39) | 3.4 (1.1) | 5.0 (1.4) | 88/94 (94) | 71/94 (76) |
| Gu HM 2019 | 17/35 (48.6) | 13/35 (37.1) | nr | nr | 34/35 (97.1) | 28/35 (80) |
| Li Juan 2019 | 36/57 (63.2) | 24/57 (42.1) | 2.29 (0.94) | 3.82 (1.2) | 54/57 (94.7) | 43/57 (75.4) |
| Liang XM 2019 | 27/44 (61.4) | 18/44 (40.9) | nr | nr | 40/44 (90.9) | 32/44 (72.7) |
| Wang X 2019 | 76/123 (61.8) | 68/123 (55.3) | 3.62 (0.75) | 4.78 (1.06) | 116/123 (94.3) | 103/123 (83.7) |
| Fu W 2020 | nr | nr | 3.5 (0.7) | 4.7 (1.0) | nr | nr |
| Lu P 2020 | 24/44 (54.5) | 20/44 (45.5) | nr | nr | 43/44 (97.7) | 36/44 (81.8) |
| Chen D 2021 | 51/80 (63.7) | 38/80 (47.5) | 3.48 (0.54) | 4.2 (0.65) | 74/80 (92.5) | 64/80 (80) |
| Gao HY 2021 | 30/46 (65.2) | 21/46 (45.6) | 2.68 (0.32) | 3.89 (0.54) | 44/46 (95.6) | 35/46 (76) |
| Ji WF 2021 | 41/65 (63.1) | 30/65 (46.1) | nr | nr | 62/65 (95.4) | 53/65 (81.5) |
| Li M 2021 | 36/42 (85.7) | 19/42 (45.2) | 1.42 (0.57) | 3.65 (1.03) | 40/42 (95.2) | 34/42 (81) |
| Xiu WS 2021 | 11/25 (44) | 9/25 (36) | 1.91 (0.56) | 3.01 (0.8) | 24/25 (96) | 19/25 (76) |
| Fan YL 2022 | 16/40 (40) | 10/40 (25) | 2.0 (0.4) | 2.9 (0.6) | 38/40 (95) | 31/40 (78) |
| Fu HB 2022 | 35/60 (58.3) | 24/60 (40) | 2.23 (0.6) | 3.16 (0.7) | 57/60 (95) | 50/60 (83.3) |
| Liu C 2022 | 45/68 (66.2) | 33/68 (48.5) | 2.03 (0.5) | 3.5 (0.6) | 65/68 (95.6) | 57/68 (83.8) |
| Xu XY 2022 | 31/40 (77.5) | 24/40 (60) | nr | nr | 38/40 (95) | 30/40 (75) |
| Zhai YJ 2022 | 17/28 (60.7) | 9/28 (32) | nr | nr | 27/28 (92.4) | 20/28 (71.4) |
| Cao RM 2023 | 25/40 (62.5) | 17/40 (42.5) | nr | nr | 38/40 (95) | 31/40 (77.5) |
| Li YW 2023 | 50/62 (80.6) | 40/62 (64.5) | 2.81 (0.52) | 4.76 (1.25) | 61/62 (98.4) | 52/62 (83.9) |
| Wang YY 2023 | 18/31 (58.1) | 8/31 (25.8) | 5.07 (1.35) | 5.81 (1.33) | 27/31 (87.1) | 20/31 (64.5) |
| Chen YC 2024 | 35/55 (63.6) | 17/55 (30.9) | 3.01 (0.6) | 4.25 (0.9) | 52/55 (94.6) | 41/55 (74.5) |

**Abbreviations**: SD, standard deviation; nr, not reported

**Supplementary Table S6**. Subgroup analysis of primary outcomes for treatment of PAGE comparing *S. boulardii* with controls

| **Groups** | **Cured (n= 54 RCTs)** | **Duration PAGE days (n= 39 RCTs)** | **TER % (n= 53 RCTs)** |
| --- | --- | --- | --- |
| Overall | RR= 1.45 (1.38, 1.53)* I^2^=6% | SMD= -1.54 (-1.79, -1.29)* I^2^=92.2% | RR=1.21 (1.18, 1.24)* I^2^=0% |
| By dose S.b.:   250 mg/d  by age group | 1.50 (1.33, 1.69)* I^2^=77.4% 1.44 (1.35, 1.53)* I^2^=0% | -2.21 (-3.65, -0.77)* I^2^=97.1% -1.47 (-1.72, -1.22)* I^2^=91.3% | 1.18 (1.10,1.27)* I^2^=0% 1.21 (1.18, 1.24)* I^2^=0% |
| By etiology:  rotaviral +  other | 1.46 (1.35,1.58)* I^2^=24.4% 1.40 (1.11,1.78)** I^2^=0% | -1.18 (-1.45, -0.90)* I^2^=86.7% -1.21 (-1.63,-0.78)*** I^2^=57.5 | 1.22 (1.18, 1.27)* I^2^=0% 1.15 (1.06, 1.24)* I^2^=0% |
| By initiation time:  <48 hours  >48 hours | 1.42 (1.31,1.54)* I^2^=0.6% 1.46 (1.35,1.57)* I^2^=25.5% | -1.68 (-2.06, -1.30)* I^2^=92.9% -1.31 (-1.64,-0.50)* I^2^=89.0% | 1.21 (1.17, 1.25)* I^2^=0% 1.22 (1.17, 1.26)* I^2^=0% |
| By type of patient:  inpatient  outpatient  mixed | 1.47 (1.39, 1.57)** I^2^=0% 1.23 (1.08, 1.41)** I^2^=0% 1.65 (1.26, 2.15** I^2^=0% | -1.59 (-1.88, -1.29)** I^2^=92.2% na na | 1.21 (1.18, 1.24)** I^2^=0% 1.14 (1.03, 1.25)** I^2^=0% 1.20 (1.10, 1.31)** I^2^==0% |

* P<0.0001 *S. boulardii* CNCM I-745 compared to controls
** P=0.005 *S. boulardii* CNCM I-745 compared to controls
*** P=0.003 *S. boulardii* CNCM I-745 compared to controls
na, not applicable, only one RCT per sub-group category

**Abbreviations**: TER, Total effectiveness rating

**Supplementary Table S7**. Secondary outcomes for treatment of PAGE comparing *S. boulardii* with controls

| **Reference** | **Number of stools/day in *S. boulardii* at study end: mean days (SD)** | **Number of stools/day in controls at study end: mean days (SD)** | **Duration of vomiting in *S. boulardii*: mean days (SD)** | **Duration of vomiting in controls: mean days (SD)** | **Length of stay, in *S. boulardii:* mean days (SD)** | **Length of stay in controls: mean days (SD)** |
| --- | --- | --- | --- | --- | --- | --- |
| Hu XY 2002 | nr | nr | 1.2 (0.4) | 1.14 (0.36) | nr | nr |
| Ji DZ 2009 | 1.74 (0.9) | 2.24 (0.9) | nr | nr | nr | nr |
| Wei WQ 2011 | nr | nr | 1.2 (0.4) | 1.14 (0.4) | nr | nr |
| Xiao QZ 2011 | nr | nr | nr | nr | nr | nr |
| Zhang JB 2011 | nr | nr | nr | nr | nr | nr |
| Fu SL 2012 | 1.43 (0.84) | 2.13 (0.9) | nr | nr | nr | nr |
| Wang DE 2013 | 2.3 (1.2) | 3.72 (1.8) | nr | nr | 3.87 (1.2) | 6.43 (1.3) |
| Zhang ZJ 2013 | nr | nr | nr | nr | 4.3 (1.4) | 5.6 (1.6) |
| Xiong B 2014 | 3.13 (0.95) | 4.15 (0.9) | nr | nr | nr | nr |
| Yuan YH 2014 | nr | nr | nr | nr | nr | nr |
| Ding L 2015 | 2.63 (1.99) | 3.98 (1.73) | nr | nr | nr | nr |
| Wu LQ 2015 | 1.95 (0.82) | 2.58 (1.29) | nr | nr | nr | nr |
| Xu ZX 2015 | 1.67 (0.9) | 2.2 (0.8) | nr | nr | nr | nr |
| Sun XL 2016 | 1.5 (0.2) | 1.54 (0.2) | 1.7 (0.3) | 2.7 (0.4) | 4.3 (1.0) | 6.7 (1.6) |
| Xia L 2016 | 1.85 (0.71) | 2.46 (0.89) | nr | nr | nr | nr |
| Yang NF 2016 | 1.1 (0.4) | 3.7 (0.6) | nr | nr | nr | nr |
| Zhao JF 2016 | nr | nr | nr | nr | nr | nr |
| Shi HJ 2017 | 1.7 (0.9) | 2.6 (0.8) | nr | nr | nr | nr |
| Yuan S 2017 | 1.38 (1.5) | 2.9 (1.6) | nr | nr | nr | nr |
| Zhang J 2017 | nr | nr | nr | nr | nr | nr |
| Zhong S 2017 | nr | nr | nr | nr | nr | nr |
| Zhou HQ 2017 | nr | nr | nr | nr | nr | nr |
| Cao Y 2018 | 2.82 (0.25) | 5.53 (0.36) | nr | nr | nr | nr |
| Chen SH 2018 | nr | nr | nr | nr | nr | nr |
| Fan XL 2018 | nr | nr | nr | nr | 4.4 (1.7) | 6.4 (1.8) |
| Jin L 2018 | 1.87 (0.95) | 2.94 (1.08) | nr | nr | nr | nr |
| Li W 2018 | 1.52 (0.66) | 3.19 (1.08) | 1.68 (0.4) | 3.35 (0.8) | 5.62 (1.48) | 7.01 (1.51) |
| Sun BJ 2018 | nr | nr | nr | nr | nr | nr |
| Sun Q 2018 | 1.62 (0.73) | 2.37 (0.89) | nr | nr | nr | nr |
| Tang QL 2018 | nr | nr | 1.6 (0.7) | 2.9 (0.7) | nr | nr |
| Wang HN 2018 | nr | nr | nr | nr | nr | nr |
| Weng DZ 2018 | nr | nr | 1.45 (0.7) | 2.13 (0.8) | nr | nr |
| Yang Ping 2018 | nr | nr | 1.78 (0.4) | 2.78 (0.3) | 5.33 (0.86) | 6.86 (1.59) |
| Yao HY 2018 | nr | nr | nr | nr | nr | nr |
| Zhang Hong 2018 | nr | nr | nr | nr | nr | nr |
| Zhao L 2018 | 2.42 (0.74) | 3.34 (0.86) | nr | nr | nr | nr |
| Gao ZH 2019 | 3.2 (0.7) | 4.4 (1.0) | nr | nr | nr | nr |
| Gu HM 2019 | 1.1 (0.45) | 3.79 (0.6) | nr | nr | nr | nr |
| Li Juan 2019 | nr | nr | 1.25 (0.84) | 1.97 (0.7) | nr | nr |
| Liang XM 2019 | nr | nr | nr | nr | nr | nr |
| Wang X 2019 | 1.78 (0.33) | 2.75 (0.42) | nr | nr | nr | nr |
| Fu W 2020 | 1.7 (0.3) | 2.4 (0.4) | nr | nr | nr | nr |
| Lu P 2020 | 1.76 (0.43) | 2.67 (0.7) | nr | nr | nr | nr |
| Chen D 2021 | 2.18 (0.74) | 2.74 (0.82) | 1.41 (0.3) | 1.86 (0.3) | 4.62 (0.5) | 6.08 (0.5) |
| Gao HY 2021 | 1.96 (0.54) | 2.36 (0.62) | 1.25 (0.2) | 1.69 (0.3) | 4.25 (0.4) | 5.89 (0.42) |
| Ji WF 2021 | nr | nr | nr | nr | nr | nr |
| Li M 2021 | 1.56 (0.5) | 3.09 (1.2) | nr | nr | nr | nr |
| Xiu WS 2021 | nr | nr | nr | nr | nr | nr |
| Fan YL 2022 | nr | nr | 1.2 (0.4) | 2.2 (0.5) | 6.1 (1.5) | 7.3 (1.0) |
| Fu HB 2022 | nr | nr | 0.98 (0.3) | 2.2 (0.6) | nr | nr |
| Liu C 2022 | nr | nr | 2.15 (0.32) | 2.83 (0.8) | 4.1 (0.45) | 5.4 (0.7) |
| Xu XY 2022 | nr | nr | nr | nr | nr | nr |
| Zhai YJ 2022 | nr | nr | nr | nr | nr | nr |
| Cao RM 2023 | nr | nr | nr | nr | nr | nr |
| Li YW 2023 | 1.07 (0.3) | 3.54 (0.5) | 1.3 (0.3) | 2.51 (0.4) | nr | nr |
| Wang YY 2023 | nr | nr | 2.34 (0.4) | 3.56 (0.6) | 5.81 (1.5) | 6.78 (1.75) |
| Chen YC 2024 | nr | nr | 1.25 (0.4) | 1.91 (0.4) | nr | nr |

**Abbreviation**: nr, not reported

**Supplementary Table S8.** Inflammatory marker outcomes for treatment of PAGE comparing *S. boulardii* with controls

| **Ref** | **Changes in TNF levels, Sb: mean pg/ml (SD)** | **Changes in TNF levels, controls: mean pg/ml (SD)** | **Change in CD4/CD8 ratio, Sb: mean (SD)** | **Change in CD4/CD8 ratio, controls: mean (SD)** | **Change in CRP,  Sb: mean mg/L (SD)** | **Change in CRP, controls: mean mg/L (SD)** | **Other type of inflamm markers  Sb vs controls** |
| --- | --- | --- | --- | --- | --- | --- | --- |
| Hu XY 2002 | nr | nr | nr | nr | nr | nr | nr |
| Ji DZ 2009 | nr | nr | nr | nr | nr | nr | nr |
| Wei WQ 2011 | nr | nr | nr | nr | nr | nr | nr |
| Xiao QZ 2011 | nr | nr | nr | nr | nr | nr | nr |
| Zhang JB 2011 | nr | nr | nr | nr | nr | nr | nr |
| Fu SL 2012 | nr | nr | nr | nr | nr | nr | nr |
| Wang DE 2013 | nr | nr | nr | nr | nr | nr | nr |
| Zhang ZJ 2013 | nr | nr | nr | nr | nr | nr | nr |
| Xiong B 2014 | nr | nr | +0.45 (0.5) | +0.15 (0.7) | nr | nr | +CD3 |
| Yuan YH 2014 | nr | nr | +0.55 (0.37) | +0.04 (0.25) | nr | nr | nr |
| Ding L 2015 | nr | nr | nr | nr | nr | nr | nr |
| Wu LQ 2015 | nr | nr | nr | nr | nr | nr | nr |
| Xu ZX 2015 | nr | nr | nr | nr | nr | nr | nr |
| Sun XL 2016 | nr | nr | -0.62 (0.3) | -0.05 (0.2) | nr | nr | +CD3 |
| Xia L 2016 | nr | nr | nr | nr | nr | nr | nr |
| Yang NF 2016 | nr | nr | nr | nr | nr | nr | nr |
| Zhao JF 2016 | nr | nr | +0.6 (0.5) | +0.07 (0.6) | nr | nr | +CD3 |
| Shi HJ 2017 | nr | nr | nr | nr | nr | nr | nr |
| Yuan S 2017 | nr | nr | +1.53 (0.5) | +0.61 (0.06) | nr | nr | +CD3 |
| Zhang J 2017 | nr | nr | nr | nr | nr | nr | nr |
| Zhong S 2017 | nr | nr | -0.43 (0.6) | +0.03 (0.9) | nr | nr | +CD3 |
| Zhou HQ 2017 | nr | nr | nr | nr | nr | nr | nr |
| Cao Y 2018 | -43.86 (6.59) | -16.29 (4.9) | nr | nr | -72.7 (14.5) | -51.2 (12.5) | - IL1, - IL6 |
| Chen SH 2018 | nr | nr | +0.74 (nr) | +0.13 (nr) | nr | nr | +CD3 |
| Fan XL 2018 | nr | nr | nr | nr | nr | nr | nr |
| Jin L 2018 | nr | nr | nr | nr | nr | nr | nr |
| Li W 2018 | nr | nr | nr | nr | nr | nr | nr |
| Sun BJ 2018 | nr | nr | nr | nr | nr | nr | nr |
| Sun Q 2018 | nr | nr | nr | nr | nr | nr | nr |
| Tang QL 2018 | nr | nr | +0.55 (0.6) | +0.06 (0.8) | nr | nr | +CD3 |
| Wang HN 2018 | nr | nr | +0.6 (0.6) | +0.2 (0.6) | nr | nr | +CD3 |
| Weng DZ 2018 | nr | nr | nr | nr | nr | nr | -IL6 |
| Yang Ping 2018 | nr | nr | nr | nr | -57.5 (3.3) | -37 (3.4) | nr |
| Yao HY 2018 | nr | nr | nr | nr | nr | nr | nr |
| Zhang Hong 2018 | -2.75 (0.8) | -1.92 (0.9) | nr | nr | nr | nr | -IL6 |
| Zhao L 2018 | nr | nr | nr | nr | nr | nr | nr |
| Gao ZH 2019 | nr | nr | nr | nr | nr | nr | nr |
| Gu HM 2019 | nr | nr | nr | nr | nr | nr | nr |
| Li Juan 2019 | -2.06 (0.38) | - 1.26 (0.17) | +0.04 (0.03) | +0.5 (0.07) | nr | nr | - IL8 |
| Liang XM 2019 | nr | nr | +0.6 (0.6) | -0.03 (0.6) | nr | nr | +CD3 |
| Wang X 2019 | nr | nr | nr | nr | nr | nr | - IL6, - PCT |
| Fu W 2020 | nr | nr | nr | nr | nr | nr | -IL6 |
| Lu P 2020 | nr | nr | nr | nr | nr | nr | nr |
| Chen D 2021 | -17.75 (2.3) | -9.5 (1.15) | nr | nr | -4.69 (0.92) | -2.87 (0.66) | -IL6, -INF-gamma |
| Gao HY 2021 | -17.5 (4.5) | -10.1 (5.6) | +0.55 (0.4) | +0.09 (0.5) | nr | nr | +CD3, - IL6 |
| Ji WF 2021 | nr | nr | +0.55 (0.3) | +0.11 (0.3) | nr | nr | +CD3 |
| Li M 2021 | nr | nr | +0.71 (2.3) | +0.12 (2.1) | nr | nr | +CD3 |
| Xiu WS 2021 | nr | nr | nr | nr | nr | nr | nr |
| Fan YL 2022 | nr | nr | nr | nr | nr | nr | nr |
| Fu HB 2022 | -13.7 (4.1) | -10.2 (4.5) | nr | nr | nr | nr | -IL6 |
| Liu C 2022 | -18.5 (3.5) | -10.9 (5.7) | nr | nr | nr | nr | +IL10, -IL13 |
| Xu XY 2022 | -18.9 (4.0) | -8.8 (4.0) | +1.42 (0.14) | +0.58 (0.1) | -6.3 (0.4) | -2.7 (0.7) | -IL6, +IgA, +IgG |
| Zhai YJ 2022 | nr | nr | nr | nr | nr | nr | nr |
| Cao RM 2023 | nr | nr | +0.55 (0.3) | +0.1 (0.3) | nr | nr | +CD3 |
| Li YW 2023 | nr | nr | nr | nr | nr | nr | nr |
| Wang YY 2023 | -2.5 (0.5) | -1.8 (0.6) | +0.69 (0.4) | +0.3 (0.4) | nr | nr | nr |
| Chen YC 2024 | -28.2 (7.2) | -1.71 (7.0) | nr | nr | -1.53 (0.4) | -0.84 (0.8) | -IL6, -INF-gamma |

**Abbreviation**: nr, not reported

**Supplementary Table S9.** GRADE assessment

| **Outcome** | **# RCT** | **# participants** | **RR/SMD** | **Risk of Bias** | **Inconsistency** | **Indirectness** | **Imprecision** | **Publication bias** | **Certainty of evidence** |
| --- | --- | --- | --- | --- | --- | --- | --- | --- | --- |
| Cured | 54 | 5453 | 1.45 (1.38, 1.53) | moderate | not serious | not serious | not serious | not serious | high |
| Duration of diarrhea | 39 | 4165 | -1.54 (-1.79, -1.29) | moderate | moderate | not serious | not serious | not serious | moderate |
| Total effectiveness rating | 53 | 5307 | 1.21 (1.18, 1.24) | moderate | not serious | not serious | not serious | not serious | high |

**Abbreviations**: #, number; RCT, randomized controlled trials; RR, relative risk; SMD, standardized mean difference

**Notes**: Risk of Bias=moderate due to lack of blinding, Inconsistency=I^2^>50% not explained by subgroups, Indirectness= limited generalizability due to participants, study design etc., Imprecision- 95% CI crossed ns line or sample size<400 participants, Publication bias, moderate is some publication bias is present, Certainty of evidence=confidence that outcome measure is consistent with other studies and overall estimate of strength of evidence.
